# Eccentric cycling is superior to standard rehabilitation for Post-ICU recovery in COVID-19 survivors

**DOI:** 10.1371/journal.pone.0340965

**Published:** 2026-02-06

**Authors:** María Fernanda Miranda, Cristian Álvarez, Jacob Earp, Álvaro N. Gurovich, Gabriel Nasri Marzuca-Nassr, Luis Peñailillo

**Affiliations:** 1 Facultad de Medicina, Clínica Alemana, Universidad del Desarrollo, Santiago, Chile; 2 Exercise and Rehabilitation Sciences Institute, School of Physical Therapy, Faculty of Rehabilitation Sciences, Universidad Andres Bello, Santiago, Chile; 3 Sport Optimization & Rehabilitation Laboratory, University of Connecticut, Storrs, Connecticut, United States of America; 4 Department of Physical Therapy and Movement Sciences, College of Health Sciences, The University of Texas at El Paso, El Paso, Texas, United States of America; 5 Departamento de Ciencias de la Rehabilitación, Facultad de Medicina, Universidad de La Frontera, Temuco, Chile; Università degli Studi di Milano: Universita degli Studi di Milano, ITALY

## Abstract

**Background:**

The COVID-19 pandemic left numerous patients with post-intensive care syndrome (PICS) that resulted in prolonged physical and health impairment. Compared to standard rehabilitation (STD REHAB), eccentric cycling (ECC) has lower cardiopulmonary demands while inducing greater functional performance improvements after training, which could be ideal for individuals with PICS.

**Aim:**

To compare the effects of eight weeks of ECC versus STD REHAB on functional performance and quality of life of patients after hospitalization.

**Methods:**

Randomised crossover design study (clinicaltrial.gov: NCT06895850). Twenty survivors of the COVID-19 infection (50.8 ± 8.8 years old) recruited six months after hospitalization were involved. Participants were initially randomized into ECC (n = 10) or STD REHAB (n = 10). Both groups trained for eight weeks, rested for two weeks, and performed the crossover with the remaining training modality. Exercise time was 20−30 min for ECC and 60 min for STD REHAB. Cardiopulmonary demand was measured during training. Body composition, whole-body manual and handgrip strength, functional performance (6-min walking test; 6MWT, timed up and go; TUG, 1-min sit to stand), cognitive (MoCa and Barthel Index), and quality of life and symptoms (QoL; Patient Health Questionnaire; PHQ and Post-COVID-19 Functional Status Scale; PCFS) were measured before and after interventions.

**Results:**

Cardiopulmonary demand during training was lesser during STD REHAB than ECC. All variables improved after both interventions, but ECC showed larger improvements in 6MWT, TUG, and Barthel Index compared to STD REHAB.

**Conclusion:**

Eccentric cycling training induced greater functional and cognitive improvements than standard rehabilitation.

## Introduction

The rapid worldwide spread of the SARS-CoV-2 coronavirus disease in 2019 (COVID-19) caused numerous hospitalizations in intensive (ICU) and intermediate care units [1]. The infection itself and the interventions, such as mechanical ventilation, sedation, and prolonged bed rest, among others, caused significant sequelae in the survivors, collectively known as Post-Intensive Care Syndrome (PICS) [2]. For instance, time in ICU and hospitalization was around 16 days after COVID-19 infection in France [3]. It has been described that sequelae from prolonged ICU hospitalization, like those observed with COVID-19, can persist for up to 5 years after discharge and can present with multisystemic impairments that result in significant physical disability and reduced quality of life [4]. Specifically, muscle atrophy, neuropathies of the extremities, neuromuscular damage (ICU-acquired weakness), cognitive impairment, and psychological deterioration have all been reported as part of the sequelae [2]. Furthermore, PICS is characterized by persistent COVID-19 symptoms after infection, including symptoms like dyspnoea, chronic cough, fatigue, and cognitive impairment [5], which can impact patients’ quality of life [6].

Rehabilitation plays a crucial role in managing PICS and regaining functional capacity [6,7]. Comprehensive rehabilitation approaches include endurance, flexibility, strength training, pulmonary rehabilitation, task-specific exercises for daily activities, psychological support, and pain management [6]. Hence, exercise-based therapy is essential but may require careful monitoring for post-exertional symptom exacerbation and orthostatic hypotension [5]. However, the rehabilitation interventions for these patients were limited due to the volume of infected individuals and the lack of knowledge about this new disease. As a result, many patients were left with severe, often disabling sequelae [8]. Further research is needed to determine optimal rehabilitation modalities in these patients [5,9].

Eccentric cycling is a novel exercise modality that has been shown to be beneficial for individuals with impaired physical or cardiopulmonary function, such as those with chronic obstructive pulmonary disease [10], heart failure [11], and older adults [12]. Compared to traditional cycling, eccentric cycling has been shown to elicit greater improvements in strength and muscle mass, achieved with relatively low metabolic and cardiopulmonary demand during training, even when shorter exercise durations are used [13]. Therefore, eccentric cycling could be beneficial for individuals with PICS, as it may enable greater tolerance to physical effort in those with cardiopulmonary, metabolic, or neuromuscular impairments [14].

Given the recent and rapid nature of the COVID-19 pandemic, there is limited evidence comparing rehabilitation modalities in people with PICS. The effectiveness of rehabilitation interventions is worth exploring in order to prescribe the best possible treatment for individuals suffering from a long period of hospitalization. Therefore, this study aimed to compare the effects of eccentric cycling (ECC) versus standard rehabilitation (STD REHAB) training on various parameters of functional performance and quality of life in patients recovering from COVID-19. It was hypothesized that ECC would induce greater functional performance and quality of life improvements compared to STD REHAB.

## Materials and methods

### Participants

**Fig 1:**
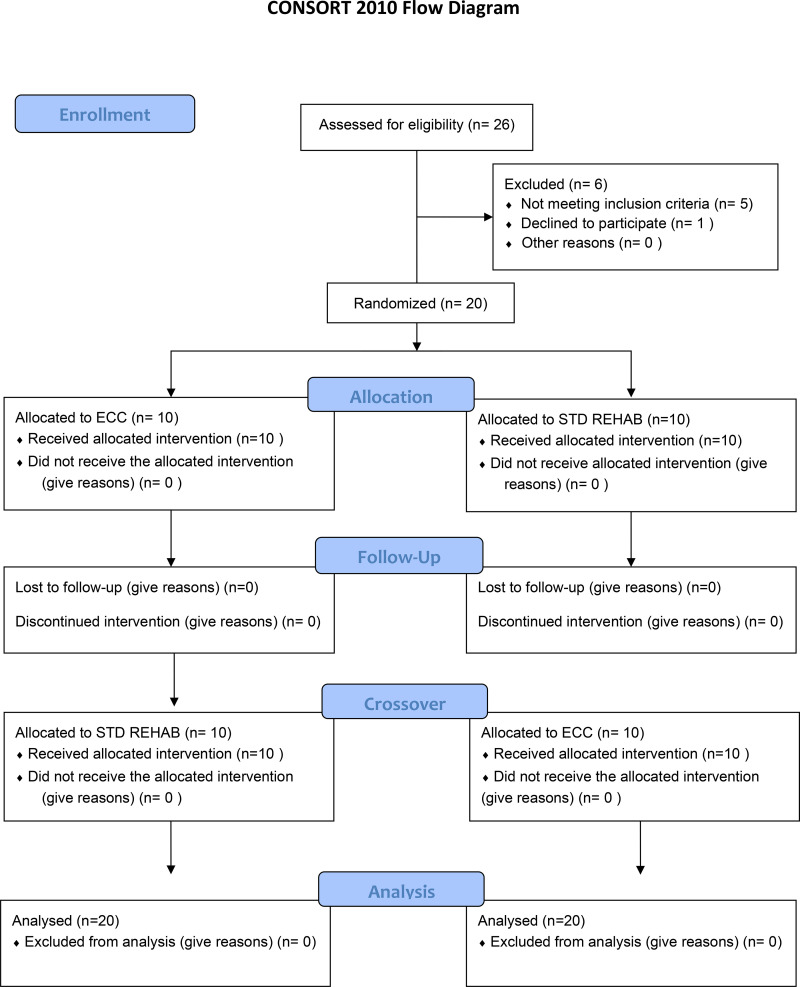
CONSORT Flow Diagram.

Twenty patients (9 men, 11 women) between the ages of 30 and 60 years who had been hospitalized for COVID-19 in an ICU volunteered for this study (Fig 1 CONSORT flow diagram). Participants were hospital-discharged at least 6 months before the start of the study and had not undergone rehabilitation after hospital discharge. Patients who did not pass the medical check-up before the study (patients with myocarditis and/or abnormal troponins or electrocardiograms from the last three months), oxygen-dependent patients, those with musculoskeletal injuries, bedridden patients, disoriented patients, or those with severe mental disabilities were excluded due to being unsafe to exercise. The baseline characteristics of the participants are described in Table 1. The participants were from a southern city of Chile (Osorno) and were recruited through posters at the hospital and in local newspapers. Recruitment started on March 1^st^, 2022, and finished on September 30, 2022. All participants signed a written informed consent approved by the Finis Terrae University Ethics Committee prior to their participation in the study. This study was registered at clinicaltrial.gov (NCT06895850).

**Table 1:** Anthropometric and demographic characteristics of the participants. Intensive care unit: ICU, Intensive treatment unit: ITU. BMI: body mass index. Means ± standard deviation.

Total participants	n = 20
Sex (%; n)	45% men (n = 9) 55% women (n = 11)
Age (years)	50.8 ± 8.8
Height (cm)	163.8 ± 8.1
Body mass (kg)	86.0 ± 16.6
BMI (kg·m^-2^)	32.5 ± 6.9
**Covid-19 Vaccination**	
No vaccines	45% (n = 9)
1 vaccine	35% (n = 7)
2 vaccines	20% (n = 4)
**Hospitalization**	
ICU (days)	14.5 ± 11.5
ITU (days)	8.7 ± 4.1
Basic hospitalization (days)	5.7 ± 2.8
Total hospitalization time (days)	28.8 ± 14.9

### Experimental design

This study utilized a two-treatment crossover design. Each treatment was eight weeks in duration, with a two-week washout period. The order of treatments was randomized and counterbalanced. Twenty participants were recruited and completed this study. This sample was deemed sufficient as an *a priori* power analysis (G*Power 3.1.9, Germany) estimated that 18 patients were required to test the anticipated effect for a crossover design, considering a statistical power of 0.8 and alpha <0.05. This effect was estimated based on an 18.7% increase in the 6-minute walking test reported in a previous study after 12 weeks of eccentric training [10]. Given a 10% dropout rate, we recruited 20 participants for this study.

**Fig 2:**
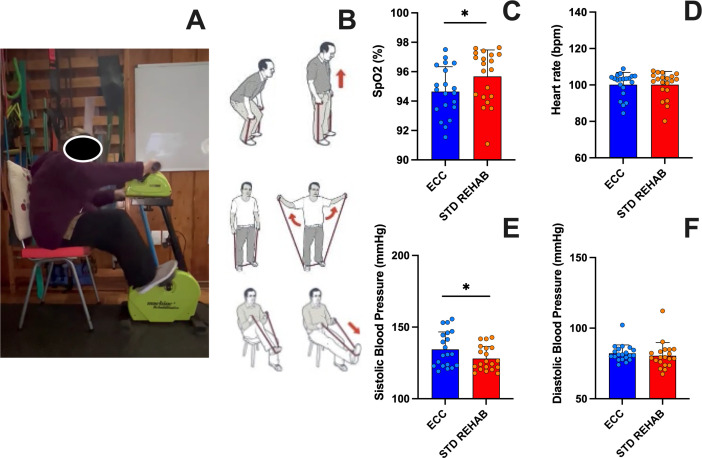
Eccentric cycling and Standard rehabilitation interventions. ECC **(A)**, STD REHAB **(B)**, average sessional oxygen saturation (SpO2; **C)**, heart rate **(D)**, systolic blood pressure **(E)**, and diastolic blood pressure (F) of eccentric cycling (ECC) and standard rehabilitation (STD REHAB) interventions. Means ± standard deviation. *: p < 0.05.

Condition order was randomly arranged as either ECC then STD REHAB sequence (n = 10) or STD REHAB then ECC sequence (n = 10) to account for a potential order effect of treatments. Both interventions lasted eight weeks, and outcome assessments were performed before and after each intervention. The post-intervention measure was considered the pre-intervention of the second intervention. As shown in Fig 2A, during the ECC participant performed eccentric cycling using an arm-and-leg motorized cycle ergometer (Model ABJ-107-2, Medical Technology Co LTD, China) while during the STD REHAB condition participants performed functional rehabilitation using strengthening exercises with elastic bands (Fig 2B). Both interventions lasted for eight weeks each (two sessions per week the first two weeks, and three session per week from week three to eight) as it was successfully used in COVID patients in a previous study [15], with a 2-week wash-out period between interventions, in which the participants refrained from training in preparation for their second intervention.

### Interventions

The exercise intensity was matched between conditions throughout the intervention period using the 6–20 point rating of perceived exertion (RPE; Borg´s Scale) [10]. For both groups, RPE progressively increased during the first two weeks from 9 (very light) to 11 (fairly light), and then over the next five weeks from 13 (somewhat hard) to 15 (hard). Additionally, heart rate (HR) and blood pressure (Omron Serie 5/7156, USA), and oxygen saturation (Heal Force A3, CHOICEMMED, China) were measured immediately after all training sessions to quantify the cardiovascular demands of training, which were averaged from all training sessions.

The training protocol for the ECC consisted of a 5-minute warm-up on a stationary concentric ergometer (RS1 Go, Life Fitness, USA) before completion of the prescribed workout. In the first two weeks, participants completed two sets of 10 minutes of eccentric cycling using upper and lower limbs at an RPE between 9–11 with 2 minutes of inter-set rest. Then, during weeks 3–8, participants completed two sets of 15 minutes of eccentric cycling at an RPE between 13–15 with 2 minutes inter-set rest as suggested by previous recommendations [14].

The training protocol for STD REHAB consisted of the same 5-minute warm-up protocol used in ECC, followed by three sets of 8 repetitions of bicep curls, triceps extensions, shoulder abduction, bodyweight squats, deadlifts, and leg abductions with an elastic band, with 2 minutes inter-set rest. The resistance of the elastic band tension was progressively increased according to the participant’s tolerance based on RPE. ECC training was programmed for ~20–30 min, while STD REHAB lasted ~60 min per session. After each training session, a verbal question was asked regarding any adverse effects that may have occurred during training.

## Primary outcomes

### Functional performance and strength

#### Muscle Strength (Medical Research Council; MRC).

Manual strength of six muscle groups (shoulder abduction, elbow flexion, wrist extension, hip flexion, knee extension, and ankle dorsiflexion) was evaluated bilaterally following the MRC scale guidelines [16]. Each muscle group was rated on a scale from 0 (paralysis) to 5 (normal strength). The MRC-sum score evaluates global muscle strength. The final score ranges from 0 (total paralysis) to 60 (normal muscle strength in all four limbs) [16].

#### Handgrip Strength.

Manual handgrip strength was evaluated with a hand dynamometer (Jamar, USA) with the participant seated with the elbow at 90°, performing a maximum grip for 3 seconds, expressed in kilograms. Three attempts were made for the dominant upper limb, with a one-minute rest between attempts, and the highest of the three values was registered [17].

#### Six-Minute Walk Test (6MWT).

The 6MWT measured the distance a person could walk in six minutes as quickly as possible. It was conducted in a 30 m long corridor. If the participant experienced chest pain, dyspnoea, sweating, cyanosis, or chest discomfort during the test, the test was stopped, and the distance covered until the onset of symptoms was recorded [18].

#### One-Minute Sit-to-Stand Test.

The participants were asked to cross their arms with hands on the opposite shoulders and to sit and stand from the chair as many times as possible in one minute [19]. This test serves as an estimate of lower extremity power and functional capacity [19].

#### Timed up and go (TUG).

Participants were instructed to rise from a chair, walk three meters, turn around a cone, walk back to the chair, and sit down with their back leaning against the backrest as quickly and safely as possible [10]. The time in seconds (s) needed to perform the entire sequence was recorded. Each participant performed the TUG in three attempts with a 2-minute rest between attempts, and the fastest time was used for further analyses.

### Quality of life and symptoms

#### Post-COVID-19 Functional Status Scale (PCFS).

The PCFS is a validated questionnaire to assess the functionality of COVID-19 survivors upon hospital discharge [20]. It consists of an interview with questions about daily living activities, instrumental activities, social activities, and lifestyle that the patient must rate from 0 (no limitations) to 4 (severe functional limitation).

## Secondary outcomes

### Body Composition

Anthropometric measurements were taken between 8:00 and 10:00 am after an overnight fast, including weight (kg) and height (cm). Furthermore, multifrequency bioimpedanciometry (InBody 120, InBody Co., Ltd, Republic of Korea) was used to determine the whole-body fat and muscle mass percentage [21] before and after interventions.

### Peak Expiratory Flow

Pulmonary function was assessed with a mini-Wright peak flow meter (Clement Clarke, Mini-Wright PFM, United Kingdom) by measuring the maximum expiratory flow maintained for 10 milliseconds, expressed in L/min. This test was performed with the participant seated with their knees and hips flexed at 90°. In this position, participants performed a maximum inspiration, placed the device’s mouthpiece in their mouth, sealed with their lips, and had their nose clipped, and then were asked to breathe out as forcefully and quickly as possible. This manoeuvre was repeated thrice with a 2-minute rest between each attempt, and the highest value achieved was recorded [22].

### Patient Health Questionnaire (PHQ-9)

The PHQ-9 is a self-administered questionnaire with nine questions about depressive symptoms, rated by how often they appear each week. The total score is grouped into the following categories: 1–9 mild or no depressive symptoms, 10–14 moderate depressive symptoms, and 15–27 severe depressive symptoms [21].

### Cognitive function and independence

#### Montreal Cognitive Assessment (MoCA).

The MoCA test assesses cognitive function and takes approximately 10 minutes [22]. The MoCA evaluates orientation, short-term memory, visuospatial and executive functioning, language skills, abstraction, animal naming, and attention aspects through a guided questionnaire. The total score is a maximum of 30 points, with an additional point added if the person has no schooling. Scores below 26 points indicate cognitive dysfunction [22].

#### Barthel index.

This instrument is widely used to assess a person’s ability to perform basic activities of daily living, providing a quantitative estimate of the degree of dependence. Patients scored their capacity to perform ten activities encompassing personal hygiene, eating, and moving around the home. Each activity was scored from 0 to 5, with 0 being unable to do it and five being completely independent in performing the task. All items were summed, with scores of 0–20 classified as total dependence, 21–60 as severe dependence, 61–90 as moderate dependence, 91–99 as slight dependence, and 100 as completely independent [23].

### Statistical analysis

All results are shown as means ± standard deviation (X ± SD). The normal data distribution was verified using the Shapiro-Wilk test and Q-Q plot analysis, and Levene´s test was performed to confirm that all assumptions were met for parametric statistics in all variables. Training monitoring variables were compared between ECC and STD REHAB using a two-way analysis of variance (ANOVA) with repeated measures, using Sidak’s test for multiple comparisons. Furthermore, the average of cardiometabolic variables during training was compared using a paired Student´s t-test. To assess any carryover effect, we performed a two-way ANOVA to determine the impact of the intervention sequence (ECC-STD REHAB or STD REHAB-ECC) on the delta of changes after each sequence and intervention. Another two-way ANOVA with repeated measures, using Sidak’s test for multiple comparisons, was used to assess the changes in absolute values between groups. Additionally, a magnitude of change analysis was conducted using the effect size (ES) calculation with Hedge’s g and a 95% confidence interval. Furthermore, to assess the within-subject effect, the delta of change for all dependent variables was compared between ECC and STD REHAB using a paired Student´s t-test. All analyses were performed using Prism 9.0 software (GraphPad, CA, USA) and SPSS version 27 (IBM Corp, USA).

## Results

### Baseline values

All participants completed the study without reporting adverse effects. The baseline anthropometric measurements are shown in Table 1. Furthermore, the absolute values at baseline and after both interventions of all outcomes are shown in Table 2. In addition, the ESs of each intervention are included in Table 2 for all variables.

**Table 2:** Before (Pre-) and after (Post-) absolute values and Two-way ANOVA results with effect sizes (ESs) for the changes observed after ECC and STD REHAB. Mean ± standard deviation (X ± SD).

	ECC					STD REHAB					Two ways ANOVA
	Pre	Post	P-value	ES	95% CI of ES	Pre	Post	P-Value	ES	95% CI of ES	Intervention x time effect
MRC-sum score (a.u.)	43.2 ± 7.6	55.2 ± 5.7	<0.001	1.76	1.03-2.49	45.1 ± 9.5	53.6 ± 6.4	<0.001	1.03	0.37-1.69	0.02
Handgrip (kg)	14.5 ± 6.2	17.9 ± 6.4	<0.001	0.52	0.11-1.15	15.4 ± 6.4	17.7 ± 6.4	<0.001	0.36	0.27-0.98	0.11
6MWT (m)	315.2 ± 117.5	454.7 ± 159.3	<0.001	0.98	0.33-1.63	296.4 ± 115.1	393.1 ± 115.3	<0.001	0.82	0.33-1.47	0.05
Sit to stand (reps)	13.0 ± 5.9	20.9 ± 7.3	<0.001	1.17	0.50-1.84	14.1 ± 5.9	20.1 ± 8.3	<0.001	0.80	0.15-1.44	0.10
TUG (s)	12.7 ± 3.7	10.5 ± 2.4	<0.001	0.71	0.07-1.35	11.9 ± 3.2	10.8 ± 2.5	0.002	0.38	0.25-1.00	0.01
PCFS (points)	3.2 ± 0.8	2.3 ± 0.7	<0.001	1.20	0.53-1.87	2.9 ± 0.9	2.5 ± 0.9	0.01	0.50	0.13-1.12	0.03
Expiratory flow (L·min^-1^)	240.3 ± 83.9	343.3 ± 99.2	<0.001	1.10	0.43-1.76	267.0 ± 70.7	347.0 ± 82.3	<0.001	1.00	0.36-1.68	0.24
Fat mass (% of BM)	39.5 ± 10.5	38.5 ± 10.0	0.03	0.10	0.53-0.71	39.1 ± 10.2	38.0 ± 10.3	0.01	0.11	0.52-0.73	0.74
Muscle mass (% of BM)	50.4 ± 8.8	51.4 ± 8.9	0.08	0.11	0.51-0.73	50.2 ± 9.1	50.4 ± 9.1	0.78	0.02	0.6-0.64	0.30
Barthel Index (points)	88.6 ± 12.8	94.5 ± 12.4	<0.001	0.46	0.17-1.08	89.9 ± 11.9	92.9 ± 11.4	<0.001	0.26	0.37-0.88	0.002
MoCA test (points)	26.1 ± 2.7	28.3 ± 2.2	<0.001	0.89	0.24-1.54	26.3 ± 3.1	27.9 ± 2.1	0.003	0.61	0.02-1.25	0.42
PHQ-9 (points)	5.4 ± 3.0	3.4 ± 3.2	<0.001	0.64	0.00-1.27	4.8 ± 3.9	3.5 ± 3.2	0.002	0.34	0.28-0.97	0.14

6MWT: six-minute walking test, TUG: Timed Up and Go test, MRC: Medical Research Council, PCFS: Post-COVID-19 Functional Status Scale, BM: Body mass, MoCA: Montreal Cognitive Assessment, PHQ-9: Patient Health Questionnaire. Hedger’s effect size (ES) references (<0.2 = negligible, 0.2–0.49 = small, 0.5–0.79 = moderate, ≥ 0.8 = large) [24], and 95% CI: 95% Confidence Interval of ES.

### Training sessions

Sessional SpO2 was 1.1% greater in the STD REHAB than in ECC (P = 0.04, Fig 2C). Post-exercise systolic blood pressure was 4.7% greater after ECC compared to STD REHAB (P = 0.007, Fig 2E). The HR and diastolic blood pressure were similar between conditions (Fig 2D and 2F).

### Respiratory flowmetry

The two-way ANOVA revealed a significant sequence x intervention effect (P = 0.04), without a main effect for sequence (P = 0.24) or intervention (P = 0.34). As shown in Table 2, the peak expiratory flow increased after ECC (52.6 ± 51.0%) and STD REHAB training (31.8 ± 15.1%) in comparison to pre-intervention values (P < 0.001). Delta of change after intervention was similar between groups (P = 0.29), and ES were large and similar between interventions (Table 2) (Data not presented in graphs).

### Body composition

The two-way ANOVA did not reveal a sequence x intervention effect (P = 0.12), nor a main effect for sequence (P = 0.75) or intervention (P = 0.74) for the percentage of body fat. As shown in Table 2, the percentage of body fat decreased after ECC training (−2.2 ± 3.1%, P = 0.03) and STD REHAB (−2.8 ± 5.7%, P = 0.01) compared to pre-intervention values. The two-way ANOVA did not reveal a sequence x intervention effect (P = 0.07), nor a main effect for sequence (P = 0.23) or intervention (P = 0.23) for the percentage of muscle mass. As shown in Table 2, the percentage of muscle mass remained similar after ECC and STD REHAB compared to pre-intervention values. The delta of change after intervention was similar between groups for percentage of muscle mass (P = 0.28) and body fat (P = 0.76). ESs for fat mass changes were large and for muscle mass were negligible and similar for both interventions (Table 2) (Data not presented in graphs).

### Functional performance and strength

**Fig 3:**
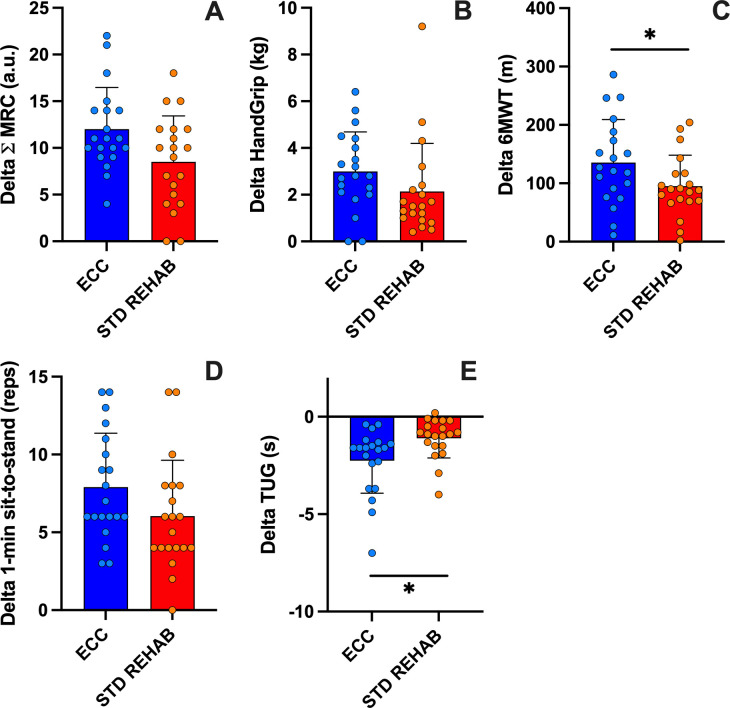
Changes in functional performance and strength. Delta of change from pre- of the sum of MRC (Medical Research Council) strength **(A)**, handgrip strength **(B)**, 6-min walking test (6MWT; **C)**, 1-min sit to stand **(D)**, and timed up and go (TUG; E) of eccentric cycling (ECC) and standard rehabilitation (STD REHAB) interventions. Means ± standard deviation. *: p < 0.05.

The two-way ANOVA did not reveal a sequence x intervention effect (P = 0.88), with a significant main effect for sequence (P = 0.001) and intervention (P = 0.01) for the sum of MRC strength. The sum of MRC strength increased after both ECC (29.9 ± 15.1%) and STD REHAB (21.4 ± 14.7%) compared to pre-intervention values (P < 0.001). The delta of change after intervention was similar between groups for the sum of MRC strength (P = 0.06; Fig 3A). The two-way ANOVA did not reveal a sequence x intervention effect (P = 0.23), or main effect for sequence (P = 0.38) or intervention (P = 0.23) for hand grip strength. Handgrip strength was significantly greater after ECC training (26.4 ± 21.9%) and STD REHAB (16.8 ± 16.6%) compared to pre-intervention values (P < 0.001). The delta of change after intervention was similar between groups for hand grip strength (P = 0.17; Fig 3B). The two-way ANOVA revealed a sequence × intervention effect (P = 0.005) and main effect for intervention (P = 0.02), without a sequence effect (P = 0.41) for the 6MWT. As shown in Table 2, the performance in the 6MWT increased after ECC (104.8 ± 236.4%) and STD REHAB (42.8 ± 48.6%) compared to pre-intervention values (P < 0.001). The delta of change after ECC was greater compared to STD REHAB for the 6MWT (P = 0.02; Fig 3C). The two-way ANOVA did not reveal a sequence x intervention effect (P = 0.83) or main effect for intervention (P = 0.11), or sequence (P = 0.90) for the one-minute sit-to-stand test. As shown in Table 2, the one-minute sit-to-stand test increased after ECC (78.6 ± 60.9%) and STD REHAB (48.9% ± 35.0%) compared to pre-intervention values (P < 0.001). The delta of change was similar between interventions for the one-minute sit-to-stand Test (P = 0.15; Fig 3D). The two-way ANOVA did not reveal a sequence × intervention effect (P = 0.31) or main effect for intervention (P = 0.29), or sequence (P = 0.31) for the TUG test. As shown in Table 2, performance in the TUG test was significantly greater after ECC (−16.5 ± 8.5%) and STD REHAB (−8.4 ± 6.2%) compared to pre-intervention values (P < 0.001). The delta of change after ECC was greater compared to STD REHAB for the 6MWT (P = 0.002; Fig 3E). The ESs for these variables were moderate to large for both interventions, but ECC showed larger ESs for TUG and handgrip strength, as shown in Table 2.

### Quality of life and symptoms

**Fig 4:**
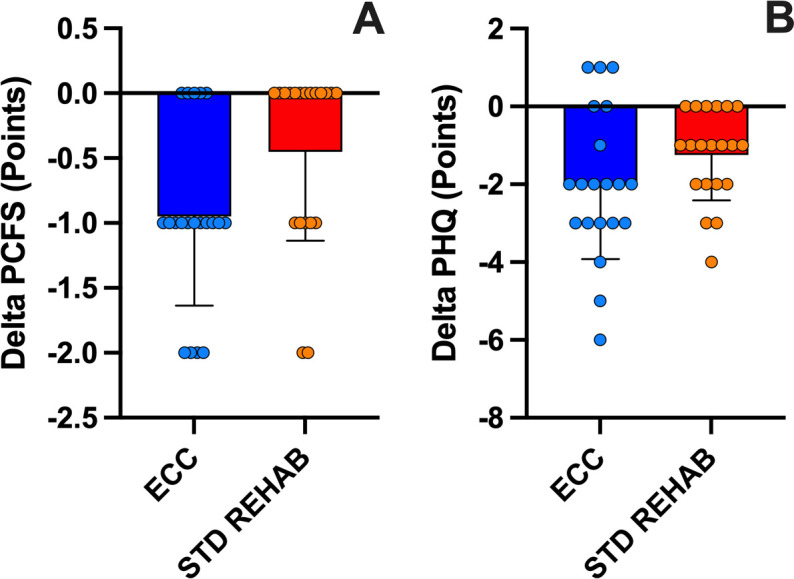
Changes in quality of life and symptoms. Delta of change from pre- of PCFS (Post-COVID-19 Functional Status Scale; A) and PHQ-9 (Patient Health Questionnaire; B) of eccentric cycling (ECC) and standard rehabilitation (STD REHAB) interventions. Means ± standard deviation. *: p < 0.05.

The two-way ANOVA did not reveal a sequence x intervention effect (P = 0.99), with a significant main effect for intervention (P = 0.03) and sequence (P = 0.01) for the PCFS. As shown in Table 2, the score on the PCFS significantly decreased after ECC (−27.5 ± 19.3%) and STD REHAB (−13.3 ± 20.5%) compared to pre-intervention values (P < 0.05). The delta of change was similar between interventions for the PCFS (P = 0.08; Fig 4A). The two-way ANOVA did not reveal a sequence x intervention effect (P = 0.37), or main effect for intervention (P = 0.15) or sequence (P = 0.28) for the PHQ-9 score. As shown in Table 2, the PHQ-9 score significantly decreased after ECC (−34.6 ± 45.2%) and STD REHAB (−26.4 ± 25.3%) compared to pre-intervention values (P < 0.01). However, the delta of change was similar between interventions for the PHQ-9 score (P = 0.14; Fig 4B). ESs for ECC were greater than STD REHAB for both variables (Table 2).

### Cognitive function and independence

**Fig 5:**
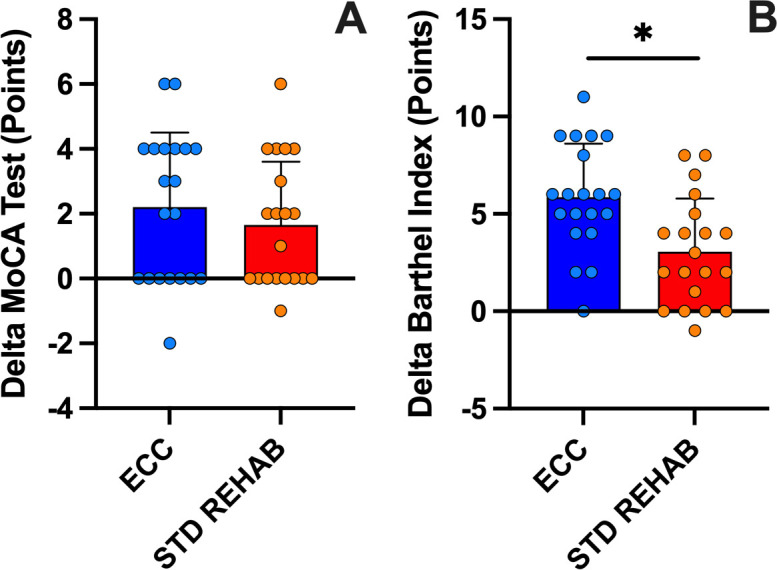
Changes in cognitive function and independence. Delta of change from pre- of MoCA (Montreal Cognitive Assessment; A) and Barthel Index (B) of eccentric cycling (ECC) and standard rehabilitation (STD REHAB) interventions. Means ± standard deviation. *: p < 0.05.

The two-way ANOVA did not reveal a sequence x intervention effect (P = 0.80), or main effect for the intervention (P = 0.38), but a significant effect of sequence (P = 0.004) for the MoCA test. As shown in Table 2, the score on the MoCA test significantly increased after ECC (9.0 ± 9.6%) and STD REHAB (7.0 ± 8.9%) compared to pre-intervention values (P < 0.01). The delta of change was similar between interventions for the MoCA (P = 0.51; Fig 5A). The two-way ANOVA did not reveal a sequence x intervention effect (P = 0.78), with a significant main effect for the intervention (P = 0.008), and sequence (P = 0.03) for the Barthel Index. As shown in Table 2, the score on the Barthel Index significantly improved after ECC (7.0 ± 3.9%) and STD REHAB (3.6 ± 3.3%) compared to pre-intervention values (P < 0.001). The delta of change was greater after ECC compared to STD REHAB for the MoCA (P = 0.02; Fig. 5B). The ES of MoCA test was greater after ECC than STD REHAB, while ES after both interventions were similar for the Barthel Index (Table 2).

## Discussion

This study aimed to compare the effects of an eccentric cycling protocol (ECC) on the functional performance and quality of life of COVID-19 survivors with a standard rehabilitation protocol consisting of strengthening exercises with elastic bands (STD REHAB). Although ECC was performed in less time per session (~20–30 min per session) than the STD REHAB (~60 min per session), it induced greater improvements in functional performance and cognitive function, while other variables improved similarly after both interventions. Therefore, the initial hypothesis is partially accepted.

Previous studies have reported that eccentric cycling training induced lesser cardiopulmonary demands than concentric or isometric training protocols to improve clinical outcomes [25, 26]. Surprisingly, our results showed that SpO_2_ and systolic blood pressure during sessions were lower during STD REHAB than ECC when training at the same perceptual intensity (11–14 on the RPE Borg´s scale) (Fig 2C-2E). This was an unexpected result, as many studies have reported a lower cardiometabolic demand for eccentric than concentric cycling [10, 24]. However, the eccentric ergometer utilized in this study involved both upper and lower extremities simultaneously, which may have required greater coordination and activation of muscles (e.g., stabilizing muscles) during cycling; hence, direct comparisons are difficult [27]. This greater muscle mass involvement and cognitive demand of ECC could increase cardiopulmonary demand, which is not typically observed in other isolated exercises such as elastic band training [28]. For instance, it has been shown that elastic band exercises are more isolated, potentially reducing cardiopulmonary demand [29]. Hence, the STD REHAB may have allowed for greater recovery during repetitions, thereby decreasing the cardiopulmonary demand compared to ECC.

We found that fat mass decreased after both interventions, while muscle mass remained unchanged (Table 2). This was somewhat unexpected as both interventions have been shown to induce increases in muscle mass in previous studies in frail populations [30]. However, a recent systematic review and meta-analysis reported inconclusive changes in muscle and fat mass after eccentric and concentric resistance training [31]. Inostroza et al. (2021) reported that 12 weeks of eccentric cycling training in patients with pulmonary disease increased fat-free mass in the lower limbs of patients with moderate Chronic Obstructive Pulmonary Disease (COPD), while concentric training was associated with a reduction in the relative fat mass of the lower limbs [10]. These discrepancies with the previous report could be explained by differences in the duration of the interventions—8 weeks in our study versus 12 weeks in the former study- and the clinical characteristics of the participants (COPD vs PICS) [32]. Gobbi et al. (2021) showed that four weeks of intervention (six sessions per week), including aerobic and strengthening exercises, induced improvements in skeletal muscle mass in patients with post-acute COVID-19 [33]. Thus, it is possible that 2–3 sessions per week for eight weeks of training were not enough to induce significant muscle mass gains in the present study. However, although no changes in muscle mass were observed, significant improvements in physical performance were evident after both interventions.

Although the time per session was lesser in ECC than in STD REHAB, our results showed that both interventions improved all functional outcomes (Table 2). However, 6WWT and TUG performance improved more after ECC than STD REHAB (Fig 3C and 3E). These results align with previous research [10] and may be attributed to greater muscle strength gains after eccentric compared to concentric exercise interventions [31]. However, the present study also measured muscle strength across different muscle groups through the MRC and handgrip strength, which increased similarly after both protocols (Fig 3A and 3B). These findings are particularly important as greater strength of the lower limbs is closely linked to improved balance and reduced risk of falls in fragile populations. These results align with previous studies demonstrating a reduction in dyspnoea and increased exercise capacity in COVID-19 survivors following a rehabilitation protocol [34].

We also evaluated the functional status using the PCFS scale, observing a decrease from moderate to severe functional limitation to mild or insignificant limitation after both interventions (Fig 4A). Furthermore, it has been shown that the PICS has a significant impact on the mental health of these patients, which has been evidenced in some studies [35]. In our study, 65% of the participants presented some depressive symptoms at the start of the study, which decreased to 20% after ECC and to 25% after STD REHAB (Table 2 and Fig 4B). These results may be explained as exercise training has shown positive impacts on chronic age-related diseases and mental health, reducing depressive and anxious symptoms [36] . In addition, both ECC and STD REHAB induced significant improvements in cognitive areas of orientation, short-term memory, visuospatial and executive functioning, language skills, abstraction, animal naming, and attention assessed in the MoCa Test (Fig 5A). The Barthel Index showed that our participants were in a slight to severe range of dependence (Table 2). Although both interventions decreased to slight dependence or independence in both groups (Table 2), the ECC showed a larger improvement in the Barthel Index after training compared to STD REHAB (Fig 5B).

This study was not free of limitations. As a prospective study, the participants’ hospitalization time before the study started differed, which could affect the initial deconditioning level of participants. Furthermore, the session duration differed between the interventions (∼30 vs 60 min), which was designed to match the ecological duration of standard rehabilitation and to follow the previous protocol recommendation [14]. Thus, clinical implications on feasibility and scalability should be considered with caution. We acknowledge that a two-week washout period may not be sufficient to allow variables to return to their pre-intervention values; however, due to ethical reasons (i.e., to avoid patient deconditioning), we implemented this washout period, which has also been used in previous studies with 6–8-week interventions [37,38]. To account for the sequence of interventions applied, we performed a two-way ANOVA analysis to assess the sequence effect between interventions. This analysis revealed that the sum of MRC strength, PCFS, MoCA, and Barthel Index variables revealed a main sequence effect. It is possible that the precision of these assessments is low (e.g., MRC), and ceiling effects are easily achieved due to the nature of the tests (e.g., questionnaires). However, even when some carryover effect was detected, we believe that the improvements induced by both interventions are comparable due to randomization. Although participants were advised not to modify their diet while participating in this study, nutrition was not monitored, which could have influenced the body composition changes. The groups were heterogeneous, with variations in age, sex, and underlying chronic diseases among the participants, which may have influenced the outcomes. High adherence is unusual in clinical studies; however, both protocols demonstrated excellent adherence (94% in ECC and 100% in STD REHAB) in these patients, with no adverse effects reported in any intervention. We speculate that high adherence to both interventions may be due to the awareness of the need for rehabilitation during the pandemic and severe deconditioning after hospitalization.

## Conclusions

In conclusion, both interventions, ECC and STD REHAB, showed large improvement in all outcomes (except muscle mass), which stresses the need for a rehabilitation protocol in PICS patients. Eccentric cycling training demonstrated greater improvements in physical and cognitive performance compared to standard rehabilitation. Furthermore, eccentric cycling could be integrated in patients rehabilitation as it requires less time and supervision. More research is needed to clinically implement eccentric cycling in patients with sequelae of long hospitalizations.

## Supporting information

S1 Checklist Consort – Consort checklist. (PDF)

S2 File Translation of project to English – Project translated to English. (DOCX)

S3 File Proyecto de tesis maria fernanda miranda final – Original Project in Spanish. (DOCX)

## References

[1] McCullough P, Kelly R, Ruocco G, Lerma E, Tumlin J, Wheelan K. Pathophysiological Basis and Rationale for Early Outpatient Treatment of SARS-CoV-2 (COVID-19) Infection. Am J Med. 2021;134(1). 10.1016/j.amjmed.2020.07.003 PMC7410805 32771461

[2] Barker-Davies RM, O’Sullivan O, Senaratne KPP, Baker P, Cranley M, Dharm-Datta S, et al. The Stanford Hall consensus statement for post-COVID-19 rehabilitation. Br J Sports Med. 2020 Aug 1;54(16):949–59. 32475821 10.1136/bjsports-2020-102596 PMC7418628

[3] Boëlle PY, Delory T, Maynadier X, Janssen C, Piarroux R, Pichenot M, et al. Trajectories of Hospitalization in COVID-19 Patients: An Observational Study in France. J Clin Med. 2020 Sept 29;9(10):3148. 33003375 10.3390/jcm9103148 PMC7600846

[4] Appleton RT, Kinsella J, Quasim T. The incidence of intensive care unit-acquired weakness syndromes: A systematic review. J Intensive Care Soc. 2015;16(2):126–36. doi: 10.1177/1751143714563016 28979394 PMC5606476

[5] Chuang HJ, Lin CW, Hsiao MY, Wang TG, Liang HW. Long COVID and rehabilitation. J Formos Med Assoc. 2024;123:S61-9. 10.1016/j.jfma.2023.03.022 PMC10101546 37061399

[6] Rathore FA, Khalil MT, Khan OJ. Rehabilitation perspectives in long COVID-19. J Pak Med Assoc. 2023;73(7):1553–5. 37469084 10.47391/JPMA.23-54

[7] Koçyiğit BF. The Role of Physical Medicine and Rehabilitation in Long Covid-19 Management. Anti-Aging East Eur. 2022;1(1):11–8.

[8] Paneroni M, Simonelli C, Saleri M, Bertacchini L, Venturelli M, Troosters T. Muscle strength and physical performance in patients without previous disabilities recovering from COVID-19 pneumonia. Am J Phys Med Rehabil. 2021;100(2):105. 33181531 10.1097/PHM.0000000000001641

[9] Pereira A, Amaral L, Dias I, Magalhães A, Abreu V, Esteves M, et al. Impact of Post-COVID-19 Condition on Health Status and Functional Capacity: A Cross-Sectional Study. Cardiopulm Phys Ther J. 2025;36(1):74.

[10] Inostroza M, Valdés O, Tapia G, Núñez O, Kompen MJ, Nosaka K, et al. Effects of eccentric vs concentric cycling training on patients with moderate COPD. Eur J Appl Physiol. 2022;122(2):489–502. doi: 10.1007/s00421-021-04850-x 34799753

[11] Chasland LC, Green DJ, Maiorana AJ, Nosaka K, Haynes A, Dembo LG, et al. Eccentric Cycling: A Promising Modality for Patients with Chronic Heart Failure. Med Sci Sports Exerc. 2017;49(4):646–51. doi: 10.1249/MSS.0000000000001151 27824689

[12] LaStayo PC, Ewy GA, Pierotti DD, Johns RK, Lindstedt S. The positive effects of negative work: increased muscle strength and decreased fall risk in a frail elderly population. J Gerontol A Biol Sci Med Sci. 2003;58(5):M419-24. doi: 10.1093/gerona/58.5.m419 12730250

[13] Lastayo PC, Reich TE, Urquhart M, Hoppeler H, Lindstedt SL. Chronic eccentric exercise: improvements in muscle strength can occur with little demand for oxygen. Am J Physiol. 1999;276(2):R611-5. doi: 10.1152/ajpregu.1999.276.2.R611 9950944

[14] Gurovich AN, Tiwari S, Kehl S, Umucu E, Peñailillo L. A novel “eccentric” therapeutic approach for individuals recovering from COVID-19. Cardiopulm Phys Ther J. 2021;32(S15).

[15] Del Valle MF, Valenzuela J, Marzuca-Nassr GN, Cabrera-Inostroza C, Del Sol M, Lizana PA, et al. Eight Weeks of Supervised Pulmonary Rehabilitation Are Effective in Improving Resting Heart Rate and Heart Rate Recovery in Severe COVID-19 Patient Survivors of Mechanical Ventilation. Medicina (Kaunas). 2022;58(4):514. doi: 10.3390/medicina58040514 35454353 PMC9028941

[16] Turan Z, Topaloglu M, Ozyemisci Taskiran O. Medical Research Council-sumscore: a tool for evaluating muscle weakness in patients with post-intensive care syndrome. Crit Care. 2020;24(1):562. 32948221 10.1186/s13054-020-03282-x PMC7499929

[17] Cronin J, Lawton T, Harris N, Kilding A, McMaster DT. A Brief Review of Handgrip Strength and Sport Performance. J Strength Cond Res. 2017;31(11):3187–217. doi: 10.1519/JSC.0000000000002149 28820854

[18] Eksombatchai D, Wongsinin T, Phongnarudech T, Thammavaranucupt K, Amornputtisathaporn N, Sungkanuparph S. Pulmonary function and six-minute-walk test in patients after recovery from COVID-19: a prospective cohort study. PLoS One. 2021;16(9):e0257040. 10.1371/journal.pone.0257040 PMC8412277 34473811

[19] Bohannon RW. Sit-to-stand test for measuring performance of lower extremity muscles. Percept Mot Skills. 1995;80(1):163–6. doi: 10.2466/pms.1995.80.1.163 7624188

[20] Klok FA, Boon GJAM, Barco S, Endres M, Geelhoed JJM, Knauss S, et al. The Post-COVID-19 Functional Status scale: a tool to measure functional status over time after COVID-19. Eur Respir J. 2020;56(1):2001494. doi: 10.1183/13993003.01494-2020 32398306 PMC7236834

[21] Cheng KY-K, Chow SK-H, Hung VW-Y, Wong CH-W, Wong RM-Y, Tsang CS-L, et al. Diagnosis of sarcopenia by evaluating skeletal muscle mass by adjusted bioimpedance analysis validated with dual-energy X-ray absorptiometry. J Cachexia Sarcopenia Muscle. 2021;12(6):2163–73. doi: 10.1002/jcsm.12825 34609065 PMC8718029

[22] Nunn AJ, Gregg I. New regression equations for predicting peak expiratory flow in adults. BMJ. 1989;298(6680):1068–70. doi: 10.1136/bmj.298.6680.1068 2497892 PMC1836460

[23] Baader M T, Molina F JL, Venezian B S, Rojas C C, Farías S R, Fierro-Freixenet C, et al. Validación y utilidad de la encuesta PHQ-9 (Patient Health Questionnaire) en el diagnóstico de depresión en pacientes usuarios de atención primaria en Chile. Rev chil neuro-psiquiatr. 2012;50(1):10–22. doi: 10.4067/s0717-92272012000100002

[24] Lakens D. Calculating and reporting effect sizes to facilitate cumulative science: a practical primer for t-tests and ANOVAs. Front Psychol. 2013;4:863. doi: 10.3389/fpsyg.2013.00863 24324449 PMC3840331

[25] Smith T, Gildeh N, Holmes C. The Montreal Cognitive Assessment: validity and utility in a memory clinic setting. Can J Psychiatry. 2007;52(5):329–32. doi: 10.1177/070674370705200508 17542384

[26] Cid-Ruzafa J, Damián-Moreno J. Valoración de la discapacidad física: el indice de Barthel. Rev Esp Salud Pública. 1997;71(2):127–37. 9546856

[27] Barreto RV, de Lima LCR, Borszcz FK, de Lucas RD, Denadai BS. Chronic adaptations to eccentric cycling training: a systematic review and meta-analysis. Int J Environ Res Public Health. 2023;20(4). 10.3390/ijerph20042861 PMC9957439 36833557

[28] Penailillo L, Santander M, Zbinden-Foncea H, Jannas-Vela S. Metabolic Demand and Indirect Markers of Muscle Damage After Eccentric Cycling With Blood Flow Restriction. Res Q Exerc Sport. 2020;91(4):705–12. doi: 10.1080/02701367.2019.1699234 32023184

[29] Nickel R, Troncoso F, Flores O, Gonzalez-Bartholin R, Mackay K, Diaz O, et al. Physiological response to eccentric and concentric cycling in patients with chronic obstructive pulmonary disease. Appl Physiol Nutr Metab. 2020;45(11):1232–7. doi: 10.1139/apnm-2020-0149 32413271

[30] Hill M, Puddiford M, Talbot C, Price M. The validity and reproducibility of perceptually regulated exercise responses during combined arm + leg cycling. Eur J Appl Physiol. 2020;120(10):2203–12. doi: 10.1007/s00421-020-04444-z 32710290

[31] Babiloni-Lopez C, Gene-Morales J, Saez-Berlanga A, Ramirez-Campillo R, Moreno-Murcia JA, Colado JC. The Use of Elastic Bands in Velocity-Based Training Allows Greater Acute External Training Stimulus and Lower Perceived Effort Compared to Weight Plates. Int J Environ Res Public Health. 2022;19(24):16616. doi: 10.3390/ijerph192416616 36554498 PMC9779371

[32] de Lima FF, Cavalheri V, Silva BSA, Grigoletto I, Uzeloto JS, Ramos D, et al. Elastic Resistance Training Produces Benefits Similar to Conventional Resistance Training in People With Chronic Obstructive Pulmonary Disease: Systematic Review and Meta-Analysis. Phys Ther. 2020;100(11):1891–905. doi: 10.1093/ptj/pzaa149 32750124

[33] Gobbi M, Bezzoli E, Ismelli F, Trotti G, Cortellezzi S, Meneguzzo F. Skeletal Muscle Mass, Sarcopenia and Rehabilitation Outcomes in Post-Acute COVID-19 Patients. J Clin Med. 2021;10(23):5623. 34884325 10.3390/jcm10235623 PMC8658326

[34] Farthing JP, Chilibeck PD. The effects of eccentric and concentric training at different velocities on muscle hypertrophy. Eur J Appl Physiol. 2003;89(6):578–86. 12756571 10.1007/s00421-003-0842-2

[35] Daher A, Balfanz P, Cornelissen C, Müller A, Bergs I, Marx N, et al. Follow up of patients with severe coronavirus disease 2019 (COVID-19): Pulmonary and extrapulmonary disease sequelae. Respir Med. 2020;174:106197. doi: 10.1016/j.rmed.2020.106197 33120193 PMC7573668

[36] Xue P, Du X, Kong J. Age-dependent mechanisms of exercise in the treatment of depression: a comprehensive review of physiological and psychological pathways. Front Psychol. 2025;16:1562434. 10.3389/fpsyg.2025.1562434 PMC12043869 40313907

[37] Banerjee P, Caulfield B, Crowe L, Clark AL. Prolonged electrical muscle stimulation exercise improves strength, peak VO2, and exercise capacity in patients with stable chronic heart failure. J Card Fail. 2009;15(4):319–26. doi: 10.1016/j.cardfail.2008.11.005 19398080

[38] Carrillo BJP, Cope E, Gurel S, Traslosheros A, Kenny A, Michot-Duval O, et al. Morning exercise and pre-breakfast metformin interact to reduce glycaemia in people with type 2 diabetes: a randomized crossover trial. J Physiol. 2024;602(23):6491–506. doi: 10.1113/JP285722 38522033 PMC11607888

