# Supplementary material for: Eccentric cycling is superior to standard rehabilitation for Post-ICU recovery in COVID-19 survivors
Source: PLoS One. 2026 Feb 6;21(2):e0340965. doi: 10.1371/journal.pone.0340965 (PMC12880636; doi:10.1371/journal.pone.0340965)
Supplement: S1 Checklist — (PDF) [file pone.0340965.s001.pdf]

CONSORT 2010 checklist of information to include when reporting a randomised trial*

Section/Topic	Item No.	Checklist item	Reported on Page No.
**Title and abstract**	1a	Identification as a randomized trial in the title	Not reported
	1b	Structured summary of trial design, methods, results, and conclusions	Page 1
**Introduction**	2a	Scientific background and explanation of rationale	Page 2
	2b	Specific objectives or hypotheses	Page 3
**Methods**	3a	Description of trial design (such as parallel, factorial), including allocation ratio	Page 4
	3b	Important changes to methods after trial commencement (such as eligibility criteria), with reasons	Not reported
	4a	Eligibility criteria for participants	Page 4
	4b	Settings and locations where the data were collected	Page 4
	5	The interventions for each group with sufficient details to allow replication, including how and when they were administered	Page 5
	6a	Completely defined pre-specified primary and secondary outcome measures, including how and when they were assessed	Page 6
	6b	Any changes to trial outcomes after the trial commenced, with reasons	Not reported
	7a	How sample size was determined	Page 5
	7b	When applicable, explanation of any interim analyses and stopping guidelines	Not reported
**Randomization**	8a	Method used to generate the random allocation sequence	Page 5

	8b	Type of randomization; details of any restriction (such as blocking and block size)	Page 5
	9	Mechanism used to implement the random allocation sequence (e.g., sequentially numbered containers), describing any steps taken to conceal the sequence until interventions were assigned	Not reported
	10	Who generated the random allocation sequence, who enrolled participants, and who assigned participants to interventions	Page 5
**Blinding**	11a	If done, who was blinded after assignment to interventions (e.g., participants, care providers, those assessing outcomes) and how	Not reported
	11b	If relevant, description of the similarity of interventions	Page 5
**Statistical methods**	12a	Statistical methods used to compare groups for primary and secondary outcomes	Page 7
	12b	Methods for additional analyses, such as subgroup analyses and adjusted analyses	Page 7
**Results**	13a	For each group, the numbers of participants who were randomly assigned, received intended treatment, and were analyzed for the primary outcome	Page 8
	13b	For each group, losses and exclusions after randomization, together with reasons	Not reported
	14a	Dates defining the periods of recruitment and follow-up	Page 4
	14b	Why the trial ended or was stopped	Not reported
	15	A table showing baseline demographic and clinical characteristics for each group	Page 8 (Table 1)
	16	For each group, number of participants (denominator) included in each analysis and whether the analysis was by original assigned groups	Page 9 (Table 2)

	17a	For each primary and secondary outcome, results for each group, and the estimated effect size and its precision (such as 95% confidence interval)	Page 10 (Tables 2 and Figures)
	17b	For binary outcomes, presentation of both absolute and relative effect sizes is recommended	Not applicable
	18	Results of any other analyses performed, including subgroup analyses and adjusted analyses, distinguishing pre-specified from exploratory	Page 10
	19	All important harms or unintended effects in each group	Not reported
**Discussion**	20	Trial limitations, addressing sources of potential bias, imprecision, and, if relevant, multiplicity of analyses	Page 12
	21	Generalizability (external validity, applicability) of the trial findings	Page 12
	22	Interpretation consistent with results, balancing benefits and harms, and considering other relevant evidence	Page 13
**Other information**	23	Registration number and name of trial registry	Page 6
	24	Where the full trial protocol can be accessed, if available	Not reported
	25	Sources of funding and other support (such as supply of drugs), role of funders	Page 1
