# Supplementary material for: Eccentric cycling is superior to standard rehabilitation for Post-ICU recovery in COVID-19 survivors
Source: PLoS One. 2026 Feb 6;21(2):e0340965. doi: 10.1371/journal.pone.0340965 (PMC12880636; doi:10.1371/journal.pone.0340965)
Supplement: S2 File — (DOCX) [file pone.0340965.s002.docx]

**RESEARCH PROJECT**

**I - PRESENTATION**

**Project Title:**
“Effects of eccentric training on the functionality and quality of life in patients post-hospitalization for COVID-19 in critical care units: a pilot study”

**Name:**
**Student:**
Maria Fernanda Miranda Núñez

**Phone:**
9 34589032

**Email:**
miranda.mariafernanda@gmail.com

**Disciplinary Area:**
Rehabilitation
Exercise Sciences ☑️

**Supervising Professor:**
Mauricio Venegas

**III – RESEARCH PROPOSAL**

**Project Abstract**

*(Max 500 words. Font: Arial, Size: 11, Line spacing: 1.5)*

In December 2019, Chinese health authorities reported an infectious respiratory disease caused by a new coronavirus (SARS-CoV-2), which rapidly spread worldwide, resulting in the ongoing pandemic. The respiratory infection caused by this virus has been named COVID-19 (COronaVIrus Disease 2019). In most cases, it causes mild or even asymptomatic illness, but about 20% of affected individuals require hospitalization with varying levels of severity. Critically ill patients may remain in intensive care units (ICU) for weeks or even months. Acute interventions used to treat these patients—such as mechanical ventilation, sedation, and prolonged bed rest—cause significant sequelae collectively known as “Post-Intensive Care Syndrome” (PICS). The consequences of a critical care stay can last up to 5 years post-discharge and are multisystemic, affecting motor function (muscle atrophy, upper and lower limb neuropathies, ICU-acquired weakness), cognitive performance, and psychological well-being, leading to considerable disability in survivors.

Given the average ICU stay with mechanical ventilation for severe COVID-19 patients is around 21 days, a significant impact on all dimensions of survivors’ quality of life is expected.

As COVID-19 is a new disease, the rehabilitation needs of severely ill survivors can only be anticipated based on data from the general population that has received critical care and survivors of similar viruses like SARS and MERS. However, there is limited literature on rehabilitation for these patients.

There is ample evidence on the benefits of eccentric training in conditions such as Chronic Obstructive Pulmonary Disease (COPD), heart failure, stroke, Parkinson’s disease, and in older adults. This type of training is characterized by greater gains in strength and muscle mass with only 50% of the metabolic cost, resulting in greater physical effort tolerance in patients.

This project proposes an eccentric rehabilitation protocol using an arm and leg cycle ergometer for survivors of severe COVID-19. It is expected to induce greater strength gains, improve physical capacity, impact cognitive and mental health sequelae, and contribute to an overall improvement in the quality of life of severe COVID-19 survivors compared to a standard kinesiological rehabilitation program.

**General Objective**

*(Font: Arial, Size: 11, Line spacing: 1.5)*

To determine the effect of an eccentric rehabilitation protocol on physical recovery and quality of life in patients who survived COVID-19 and were hospitalized in critical care units, and to compare it with a standard kinesiology rehabilitation protocol.

**Specific Objectives**

*(Max 1 page. Font: Arial, Size: 11, Line spacing: 1.5)*

- To determine changes in functional status using the Post COVID-19 Functional Status Scale and compare outcomes between eccentric training and a standard kinesiology rehabilitation protocol.
- To determine and compare changes in quality of life between eccentric training and a standard kinesiology rehabilitation protocol.
- To determine and compare changes in body composition (muscle mass and fat) through anthropometric measurements before and after eccentric training and a standard rehabilitation protocol.
- To determine and compare the level of dependency using the Barthel Index before and after both protocols.
- To identify which protocol is more effective in improving symptoms of dyspnea and fatigue.
- To determine and compare cognitive capacity changes using the MoCA test between both training approaches.
- To compare strength gains in upper and lower limbs using eccentric training versus standard rehabilitation.
- To determine the risk of falls and balance outcomes between patients undergoing eccentric training and those following the standard rehabilitation protocol.
- To determine and compare changes in peak expiratory flow using a Wright peak flow meter in both groups.

**Theoretical Foundations**

*(Max 3000 words. Font: Arial, Size: 11, Line spacing: 1.5)*

On December 31, 2019, an alert was issued in Wuhan, China, about the emergence of a new virus causing pneumonia and catastrophic respiratory failure, which quickly spread worldwide (World Health Organization, 2020). As of July 2021, over 60 million infections and more than 2.1 million deaths were recorded in 189 countries (Johns Hopkins University). In Chile, 1,650,483 total cases and 32,973 deaths were documented (DEIS, MINSAL).

The new coronavirus, SARS-CoV-2, is transmitted person-to-person and has high infectivity. It causes a wide range of symptoms, from upper respiratory issues, skin lesions, and gastrointestinal symptoms, to loss of taste and smell, and severe respiratory distress with systemic inflammation—leading to thrombotic events, vasculitis, myocarditis, and other complications (McCullough et al., 2021). Around 80% of patients develop mild multi-symptomatic illness, while the remaining 20% develop bilateral interstitial pneumonia that leads to respiratory failure (Weiss & Murdoch, 2020; McCullough et al., 2021).

The virus enters the body by binding to the angiotensin-converting enzyme 2 (ACE2), which is present in various organs such as the kidneys, lungs, heart, nasal mucosa, liver, blood vessels, immune system, skin, skeletal muscles, and brain (Hoffmann et al., 2020). This widespread expression explains the disease's diverse clinical manifestations. The virus also triggers a dysregulated immune response known as a “cytokine storm,” responsible for much of the clinical deterioration (Chen et al., 2020).

Mechanical ventilation combined with sedation, muscle relaxants, vasoactive drugs, prolonged immobility, and prone positioning during ICU care cause numerous complications, collectively called **Post-Intensive Care Syndrome (PICS)**. These impairments extend beyond ICU discharge and can last 5–15 years (Desai et al., 2011). PICS includes:

1. **Psychological complications:**
   Anxiety affects up to 48% of ICU survivors (Mazza et al., 2020), depression up to 30%, and post-traumatic stress disorder (PTSD) between 10–50% (Hodgson, 2017).
2. **Cognitive complications:**
   ICU-related brain dysfunction often presents as delirium. After discharge, cognitive impairments persist—especially in attention, memory, processing speed, and executive function. Studies show cognitive deficits in 20–80% of ICU survivors (Pandharipande et al., 2013).
3. **Physical complications:**
   These are pulmonary and extrapulmonary. Pulmonary issues include restrictive patterns from fibrosis and respiratory muscle weakness (Levine et al., 2008; Fan et al., 2014), further worsened by COVID-19-induced lung fibrosis (McDonald, 2021). Extrapulmonary effects include ICU-acquired weakness (ICU-AW), defined as acute symmetrical muscle weakness in both upper and lower limbs due to critical illness (Lad et al., 2020; Latronico et al., 2011). Its incidence is around 40% in critically ill adults (Appleton, 2014). Causes include catabolism, immobility, and microvascular injuries from vasoactive drugs. Since ACE2 is expressed in muscle cells, they are also directly affected by the virus (Yamamoto et al., 2020). ICU-AW is linked to sepsis, multi-organ failure, systemic inflammation, long-term mechanical ventilation, immobility, hyperglycemia, corticosteroids, and neuromuscular blockers—all commonly seen in COVID-19 patients (Appleton, 2015).

PICS is now recognized as a public health concern due to the associated disability. Its true prevalence remains uncertain due to underdiagnosis. In non-pandemic times, PICS affected 30–50% of ICU survivors (Delgado, 2017; Beaudart et al., 2018). One-third of these patients never return to work; another third return to lower-paying or different jobs (Griffiths, 2013).

Among PICS components, ICU-AW is strongly tied to decreased quality of life, lower physical capacity, and increased morbidity and mortality (Fan, 2014). It affects 26–65% of patients on mechanical ventilation for over five days and is directly correlated with days ventilated (Levine et al., 2008). In a study of SARS survivors who required ventilation (Tansey et al., 2007), all reported weakness at discharge, and 37% continued to experience significant physical impairment one year later. Hui et al. (2005) showed 6-minute walk test performance remained significantly reduced one year after ICU discharge.

In a cross-sectional study of COVID-19 pneumonia survivors, Paneroni et al. (2021) found quadriceps strength at 54% and biceps at 69% of expected normal values. The 1-minute sit-to-stand test (1minSTST) showed a 63% performance compared to norms, with 24% experiencing exercise-induced desaturation.

In another study, 85% of 118 patients who required ventilation were dependent at discharge, compared to only 6% at admission (Musheyev et al., 2021). These data highlight the urgent need for rehabilitation programs. Other long-term symptoms include fatigue, dry cough, exertional dyspnea, palpitations, dizziness, loss of balance, headaches, hair loss, and sensory disturbances (Mandal et al., 2020; Dani et al., 2021).

**Motor rehabilitation** is a key strategy for recovery after critical illness (Needham et al., 2012; Kress et al., 2014; Colbenson et al., 2019). Post-COVID-19 PICS requires multidisciplinary care, with exercise-based therapies at its core. These can improve physical function and potentially benefit emotional and cognitive health while addressing persistent symptoms.

Rehabilitation societies stress the need to implement programs for the post-acute phase of illness and improve access to services. However, during the pandemic, resources have primarily focused on critically ill patients, neglecting post-acute recovery.

A **simple and effective rehabilitation program** is urgently needed—one that is both easy for healthcare professionals to administer and for patients to follow.

Gurovich et al. (2021) proposed eccentric training as a therapeutic alternative to improve physical and mental health in post-COVID-19 patients, based on existing evidence in clinical populations. Mitchell et al. (2017) also suggested eccentric training as ideal for ICU survivors.

**Eccentric training** involves muscle actions where the muscle lengthens under tension (e.g., walking downhill or descending stairs), unlike concentric (shortening) or isometric (static) contractions. These tasks feel "easier" but provide the strongest stimulus for muscle strength and mobility gains with low metabolic cost (Lastayo et al., 1999; 2003).

Eccentric exercise is a novel approach that improves muscle strength and mass at lower energy expenditure than traditional aerobic training (Peñailillo et al., 2014). It is well-tolerated by patients with chronic diseases such as COPD (Nickel et al., 2020), heart failure (Casillas et al., 2016), coronary artery disease, type 2 diabetes, cancer survivors, Parkinson’s disease, multiple sclerosis, and stroke.

Given its advantages and compatibility with limited-exertion tolerance in COVID-19 survivors, **eccentric cycle ergometer training** may be an ideal rehabilitation method.

Therefore, this study proposes an **8-week pilot intervention** to compare the effectiveness of eccentric training versus a standard rehabilitation protocol (based on guidelines from rehabilitation societies) in improving functionality and quality of life in survivors of severe COVID-19.

**Methodology**

*(Max 3000 words. Font: Arial, Size: 11, Line spacing: 1.5)*

A prospective, comparative, randomized study will be conducted with volunteer patients who were hospitalized for COVID-19 in an intensive care unit (ICU) within the last 6 months and who have not undergone any rehabilitation after hospital discharge.

A total of 20 volunteer patients from Osorno and nearby areas (Los Lagos Region, Chile) will be included after signing an informed consent form. Participants will be recruited via posters (Annex I) in hospitals, social media, and local newspapers. The sample size was based on changes in muscle mass observed in a prior study, which showed a 4.5% increase in muscle mass in COPD patients after 12 weeks of eccentric training (Peñailillo et al., 2021, In Press). The calculation considered an alpha level of 0.05, 80% statistical power, and P < 0.05.

**Inclusion Criteria:**

- Participants aged 30–60 years.
- All patients will undergo a general medical check-up (Annex II) performed by the lead researcher.

**Exclusion Criteria:**

- Patients who fail to complete initial, intermediate, or final measurements.
- Patients unfit according to medical screening (e.g., myocarditis, abnormal troponins or ECGs in the past 3 months) (Alderighi et al., 2020; Mahumud et al., 2020; McKinney et al., 2020; Agustine et al., 2021).
- Oxygen-dependent patients or those with musculoskeletal injuries preventing physical activity.
- Patients with contraindications to exercise, disorientation, severe mental disability, or who are bedridden.
- Patients unable to tolerate the first familiarization session.

All participants must sign the informed consent.

**Intervention Protocol:**

Both groups will undergo an 8-week intervention, with measurements taken before and after. During training sessions, vital signs will be monitored: blood pressure, oxygen saturation, heart rate, respiratory rate, limb muscle pain (using Visual Analog Scale – Annex III), and dyspnea (using the MRC Dyspnea Scale – Annex IV), before and after each session.

Participants will be randomly assigned to two groups:

- **ECC Group (n = 10):** Eccentric arm and leg cycling on a cycle ergometer (Annex V).
- **STD Group (n = 10):** Standard rehabilitation based on interdisciplinary COVID-19 rehab consensus, including concentric cycle ergometer use and resistance exercises with elastic bands and/or bodyweight for upper and lower limbs (Annex VI).

Each group will have a familiarization session with equipment, exercises, scales, and assessment of post-session discomfort.

**Session Frequency:**

- Weeks 1–2: 2 sessions/week (spaced 2 days apart).
- Weeks 3–8: 3 sessions/week (spaced 1 day apart).

**Exercise Intensity:**

Intensity will be measured using the **Borg Rating of Perceived Exertion (RPE)** scale (6–20) where 6 = no exertion at all and 20 = maximal exertion (Annex VII). This scale is validated for clinical populations, athletes, children, and the elderly (Coquart et al., 2012).

**Warm-Up:**
Both groups will perform 5 minutes of stretching and activation exercises.

**ECC Group Training:**

- Weeks 1–2: 2 cycles of 10 minutes each, with 2-minute rest between, RPE 9–11.
- Weeks 3–8: 2 cycles of 15 minutes, 2-minute rest between, RPE 13–15.

**STD Group Training:**

- 5-minute cycling warm-up at RPE 6–10.
- 3 sets of 8 reps of elastic band exercises (moderate resistance, RPE 9–11), with 2-minute rests between sets. Exercises target both upper and lower limbs (e.g., chest fly, row, squats, deadlifts).

**Session Postponement Criteria:**

If any of the following are present, the session will be postponed:

- Intense dyspnea not relieved by rest
- Chest pain or tightness
- Difficulty breathing
- Intense coughing
- Dizziness
- Blurred vision
- Resting heart rate > 120 bpm
- Blood pressure < 80/60 mmHg or > 150/100 mmHg
- Oxygen saturation < 90% before exercise
- Fever or suspected reinfection symptoms

**Measurements Before and After 8-Week Intervention:**

1. **Muscle Strength (Medical Research Council Scale – Annex VIII)**
2. **6-Minute Walk Test (Annex IX)**
3. **1-Minute Sit-to-Stand Test (Annex X)**
4. **Activities of Daily Living (Barthel Index – Annex XI)**
5. **Post COVID-19 Functional Status Scale (Annex XII)**
6. **Depressive Symptoms (PHQ-9 Questionnaire – Annex XIII)**
7. **Body Composition (InBody 120, using Lookin’Body120 software)**
8. **Peak Expiratory Flow (Mini Wright Peak Flow Meter – Annex XIV)**
9. **Grip Strength (JAMAR Dynamometer – Annex XV)**
10. **Dyspnea (Modified MRC Scale – Annex IV)**
11. **Fall Risk (Timed Up and Go Test)**
12. **Balance (Berg Balance Scale – Annex XVI)**
13. **Fatigue (Brief Fatigue Inventory – BFI – Annex XVII)**
14. **Cognitive Function (Montreal Cognitive Assessment – MoCA – Annex XVIII)**

**Location and Materials:**

The study will take place at **Nexus Rehabilitation Center** in Osorno. The center’s director, Juan Eduardo Villagra, signed the authorization letter (Annex XIX).

**Statistical Analysis Plan:**

A two-way repeated measures ANOVA will be used to compare pre- and post-intervention results across two groups. Data processing will use Microsoft Excel and **SPSS (Statistical Package for the Social Sciences)**.

**References**

(Mínimo 30 referencias, estilo Vancouver, Letra Arial – Tamaño 11)

Appleton, R. T. D., Kinsella, J., & Quasim, T. (2015). The incidence of intensive care unit-acquired weakness syndromes: A systematic review. *Journal of the Intensive Care Society*, *16*(2), 126– 136. https://doi.org/10.1177/1751143714563016

Arbour, N., Day, R., Newcombe, J., & Talbot, P. J. (2000). Neuroinvasion by Human Respiratory Coronaviruses. *Journal of Virology*, *74*(19), 8913–8921. https://doi.org/10.1128/jvi.74.19.8913- 8921.2000

Baader M, Tomas, Molina F, José Luis, Venezian B, Silvia, Rojas C, Carmen, Farías S, Renata, Fierro-Freixenet, Carlos, Backenstrass, Mathias, & Mundt, Christoph. (2012). Validación y utilidad de la encuesta PHQ-9 (Patient Health Questionnaire) en el diagnóstico de depresión en pacientes usuarios de atención primaria en Chile. *Revista chilena de neuro psiquiatría*, 50(1), 22. https://dx.doi.org/10.4067/S0717-92272012000100002

Bauer, J., Vincent, I., Buckenmeyer, P., Sutherlin, M., Lind, E., Dames, K., Generali, A., Schrom, B., & Mills, M. (2017). Parkinson’s Disease: Eccentric Training To Reduce Symptoms 1299 June 1 8 00 AM - 8 20 AM. *Medicine & Science in Sports & Exercise*, *49*(5S), 352. https://doi.org/10.1249/01.mss.0000517843.91267.4a

Beaudart, C., Biver, E., Bruyère, O., Cooper, C., Al-Daghri, N., Reginster, J. Y., & Rizzoli, R. (2018). Assessment of Quality of Life in Musculo-Skeletal Health Europe PMC Funders Group. *Aging Clin Exp Res*, *30*(5), 413–418. https://doi.org/10.1007/s40520-017-0794-8.Assessment

Bohannon R. W. (1995). Sit-to-stand test for measuring performance of lower extremity muscles. *Perceptual and motor skills*, *80*(1), 163–166. https://doi.org/10.2466/pms.1995.80.1.163

Bourgonje, A. R., Abdulle, A. E., Timens, W., Hillebrands, J. L., Navis, G. J., Gordijn, S. J., Bolling, M. C., Dijkstra, G., Voors, A. A., Osterhaus, A. D., van der Voort, P. H., Mulder, D. J., & van Goor, H. (2020). Angiotensin-converting enzyme 2 (ACE2), SARS-CoV-2 and the pathophysiology of coronavirus disease 2019 (COVID-19). *The Journal of pathology,* 251(3), 228–248. https://doi.org/10.1002/path.5471

Butland, R. J., Pang, J., Gross, E. R., Woodcock, A. A., & Geddes, D. M. (1982). Two-, six-, and 12- minute walking tests in respiratory disease. *British medical journal (Clinical research ed.),* 284(6329), 1607–1608. https://doi.org/10.1136/bmj.284.6329.1607

Casillas, J. M., Besson, D., Hannequin, A., Gremeaux, V., Morisset, C., Tordi, N., et al. (2016). Effects of an eccentric training personalized by a low rate of perceived exertion on the maximal capacities in chronic heart failure: a randomized controlled trial. *Eur. J. Phys. Rehabil. Med.* 52, 159–168.

Celis-Morales, C. A., Welsh, P., Lyall, D. M., Steell, L., Petermann, F., Anderson, J., Iliodromiti, S., Sillars, A., Graham, N., Mackay, D. F., Pell, J. P., Gill, J., Sattar, N., & Gray, S. R. (2018). Associations of grip strength with cardiovascular, respiratory, and cancer outcomes and all cause mortality: prospective cohort study of half a million UK Biobank participants. BMJ (*Clinical research ed.*), 361, k1651. https://doi.org/10.1136/bmj.k1651

Chen, G., Wu, D., Guo, W., Cao, Y., Huang, D., Wang, H., Wang, T., Zhang, X., Chen, H., Yu, H., Zhang, X., Zhang, M., Wu, S., Song, J., Chen, T., Han, M., Li, S., Luo, X., Zhao, J., & Ning, Q. (2020). Clinical and immunological features of severe and moderate coronavirus disease 2019. *The Journal of clinical investigation,* 130(5), 2620–2629.

https://doi.org/10.1172/JCI137244

16

Cheng, S., & Wong, C. W. (2005). Psychological intervention with sufferers from severe acute respiratory syndrome (SARS): lessons learnt from empirical findings. *Clinical Psychology & Psychotherapy*, 12(1), 80–86. https://doi.org/10.1002/cpp.429

Cid-Ruzafa, Javier, & Damián-Moreno, Javier. (1997). Valoración de la discapacidad física: el indice de Barthel. *Revista Española de Salud Pública,* 71(2), 127-137. Recuperado en 20 de junio de 2021, de http://scielo.isciii.es/scielo.php?script=sci_arttext&pid=S1135- 57271997000200004&lng=es&tlng=es

Colbenson, G. A., Johnson, A., & Wilson, M. E. (2019). Post-intensive care syndrome: impact, prevention, and management. *Breathe* (Sheffield, England), 15(2), 98–101. https://doi.org/10.1183/20734735.0013-2019

Cooper, R., Kuh, D., Cooper, C., Gale, C. R., Lawlor, D. A., Matthews, F., Hardy, R., & FAL Con and HALCyon Study Teams (2011). Objective measures of physical capability and subsequent health: a systematic review. *Age and ageing*, 40(1), 14–23. https://doi.org/10.1093/ageing/afq117

Coquart, J. B., Tourny-Chollet, C., Lemaître, F., Lemaire, C., Grosbois, J. M., & Garcin, M. (2012). Relevance of the measure of perceived exertion for the rehabilitation of obese patients. *Annals of physical and rehabilitation medicine*, 55(9-10), 623–640. https://doi.org/10.1016/j.rehab.2012.07.003

Covid-19, A. P. (2020). Consenso Interdisciplinario de Rehabilitación para Personas. 1–154.

Cronin, J., Lawton, T., Harris, N., Kilding, A., & McMaster, D. T. (2017). A Brief Review of Handgrip Strength and Sport Performance. *Journal of strength and conditioning research,* 31(11), 3187–3217. https://doi.org/10.1519/JSC.0000000000002149

Dani, M., Dirksen, A., Taraborrelli, P., Torocastro, M., Panagopoulos, D., Sutton, R., & Lim, P. B. (2021). Autonomic dysfunction in 'long COVID': rationale, physiology and management strategies. *Clinical medicine* (London, England), 21(1), e63–e67.

https://doi.org/10.7861/clinmed.2020-0896

Desai, S. V., Law, T. J., & Needham, D. M. (2011). Long-term complications of critical care. *Critical care medicine*, 39(2), 371–379. https://doi.org/10.1097/CCM.0b013e3181fd66e5

Engardt, M., Knutsson, E., Jonsson, M., & Sternhag, M. (1995). Dynamic muscle strength training in stroke patients: effects on knee extension torque, electromyographic activity, and motor function*. Archives of physical medicine and rehabilitation*, 76(5), 419–425. https://doi.org/10.1016/s0003-9993(95)80570-2

Enright, P. L., & Sherrill, D. L. (1998). Reference equations for the six-minute walk in healthy adults. *American journal of respiratory and critical care medicine*, 158(5 Pt 1), 1384–1387. https://doi.org/10.1164/ajrccm.158.5.9710086

Fan, E., Cheek, F., Chlan, L., Gosselink, R., Hart, N., Herridge, M. S., Hopkins, R. O., Hough, C. L., Kress, J. P., Latronico, N., Moss, M., Needham, D. M., Rich, M. M., Stevens, R. D., Wilson, K. C., Winkelman, C., Zochodne, D. W., Ali, N. A., ATS Committee on ICU-acquired Weakness in Adults, & American Thoracic Society (2014). An official American Thoracic Society Clinical Practice guideline: the diagnosis of intensive care unit-acquired weakness in adults*. American journal of respiratory and critical care medicine,* 190(12), 1437–1446.

Fan, E., Dowdy, D. W., Colantuoni, E., Mendez-Tellez, P. A., Sevransky, J. E., Shanholtz, C., Himmelfarb, C. R., Desai, S. V., Ciesla, N., Herridge, M. S., Pronovost, P. J., & Needham, D. M. (2014). Physical complications in acute lung injury survivors: a two-year longitudinal prospective study. *Critical care medicine*, 42(4), 849–859. https://doi.org/10.1097/CCM.0000000000000040

Flann, K. L., LaStayo, P. C., McClain, D. A., Hazel, M., & Lindstedt, S. L. (2011). Muscle damage and muscle remodeling: no pain, no gain?. *The Journal of experimental biology*, 214(Pt 4), 674–679. https://doi.org/10.1242/jeb.050112

Fridén, J., Seger, J., Sjöström, M., & Ekblom, B. (1983). Adaptive response in human skeletal muscle subjected to prolonged eccentric training. *International journal of sports medicine*, 4(3), 177–183. https://doi.org/10.1055/s-2008-1026031

Griffiths, J., Hatch, R. A., Bishop, J., Morgan, K., Jenkinson, C., Cuthbertson, B. H., & Brett, S. J. (2013). An exploration of social and economic outcome and associated health-related quality of life after critical illness in general intensive care unit survivors: a 12-month follow-up study. *Critical care* (London, England), 17(3), R100. https://doi.org/10.1186/cc12745

Guo, Q., Zheng, Y., Shi, J., Wang, J., Li, G., Li, C., Fromson, J. A., Xu, Y., Liu, X., Xu, H., Zhang, T., Lu, Y., Chen, X., Hu, H., Tang, Y., Yang, S., Zhou, H., Wang, X., Chen, H., Wang, Z., … Yang, Z. (2020). Immediate psychological distress in quarantined patients with COVID-19 and its association with peripheral inflammation: A mixed method study. *Brain, behavior, and immunity*, 88, 17–27. https://doi.org/10.1016/j.bbi.2020.05.038

Gurovich, Alvaro N. PT, PhD, FACSM1; Tiwari, Sangeeta PhD2; Kehl, Stephanie MS2; Umucu, Emre PhD3; Peñailillo, Luis PT, PhD4 A Novel “Eccentric” Therapeutic Approach for Individuals Recovering From COVID-19, *Cardiopulmonary Physical Therapy Journal:* April 2021 - Volume 32 - Issue - p S15-S21 doi: 10.1097/CPT.0000000000000163

Hamilton, G. F., McDonald, C., & Chenier, T. C. (1992). Measurement of grip strength: validity and reliability of the sphygmomanometer and jamar grip dynamometer. *The Journal of orthopaedic and sports physical therapy*, 16(5), 215–219. https://doi.org/10.2519/jospt.1992.16.5.215

18

Harrison, A. J., Burdon, C. A., Groeller, H., & Peoples, G. E. (2020). The Acute Physiological Responses of Eccentric Cycling During the Recovery Periods of a High Intensity Concentric Cycling Interval Session. *Frontiers in physiology*, 11, 336. https://doi.org/10.3389/fphys.2020.00336

Herridge, M. S., Tansey, C. M., Matté, A., Tomlinson, G., Diaz-Granados, N., Cooper, A., Guest, C. B., Mazer, C. D., Mehta, S., Stewart, T. E., Kudlow, P., Cook, D., Slutsky, A. S., Cheung, A. M., & Canadian Critical Care Trials Group (2011). Functional disability 5 years after acute respiratory distress syndrome. *The New England journal of medicine,* 364(14), 1293–1304. https://doi.org/10.1056/NEJMoa1011802

Hodgson, C. L., Udy, A. A., Bailey, M., Barrett, J., Bellomo, R., Bucknall, T., Gabbe, B. J., Higgins, A. M., Iwashyna, T. J., Hunt-Smith, J., Murray, L. J., Myles, P. S., Ponsford, J., Pilcher, D., Walker, C., Young, M., & Cooper, D. J. (2017). The impact of disability in survivors of critical illness. *Intensive care medicine*, 43(7), 992–1001. https://doi.org/10.1007/s00134-017-4830-0

Hoffmann, M., Kleine-Weber, H., Schroeder, S., Krüger, N., Herrler, T., Erichsen, S., Schiergens, T. S., Herrler, G., Wu, N. H., Nitsche, A., Müller, M. A., Drosten, C., & Pöhlmann, S. (2020). SARS-CoV-2 Cell Entry Depends on ACE2 and TMPRSS2 and Is Blocked by a Clinically Proven Protease Inhibitor. *Cell,* 181(2), 271–280.e8. https://doi.org/10.1016/j.cell.2020.02.052

Honigsbaum M. (2013). "An inexpressible dread": psychoses of influenza at fin-de siècle. *Lancet* (London, England), 381(9871), 988–989. https://doi.org/10.1016/S0140- 6736(13)60701-1

Hui, D. S., Wong, K. T., Ko, F. W., Tam, L. S., Chan, D. P., Woo, J., & Sung, J. J. (2005). The 1-year impact of severe acute respiratory syndrome on pulmonary function, exercise capacity, and quality of life in a cohort of survivors. *Chest*, 128(4), 2247–2261. https://doi.org/10.1378/chest.128.4.2247

Irby, A, Gutierrez, J, Chamberlin, C, Thomas, SJ, Rosen, AB. Clinical management of tendinopathy: A systematic review of systematic reviews evaluating the effectiveness of tendinopathy treatments. *Scand J Med Sci Sports*. 2020; 30: 1810– 1826. https://doi.org/10.1111/sms.13734

Kirk-Sanchez, N. J., & McGough, E. L. (2014). Physical exercise and cognitive performance in the elderly: current perspectives. *Clinical interventions in aging*, 9, 51– 62. https://doi.org/10.2147/CIA.S39506

Klok, F. A., Boon, G., Barco, S., Endres, M., Geelhoed, J., Knauss, S., Rezek, S. A.,

Spruit, M. A., Vehreschild, J., & Siegerink, B. (2020). The Post-COVID-19 Functional Status scale: a tool to measure functional status over time after COVID-19. *The European respiratory journal,* 56(1), 2001494. https://doi.org/10.1183/13993003.01494- 2020

Kress, J. P., & Hall, J. B. (2014). ICU-acquired weakness and recovery from critical illness. *The New England journal of medicine*, 370(17), 1626–1635. https://doi.org/10.1056/NEJMra1209390

Kvam, S., Kleppe, C. L., Nordhus, I. H., & Hovland, A. (2016). Exercise as a treatment for depression: A meta-analysis. Journal of affective disorders, 202, 67–86. https://doi.org/10.1016/j.jad.2016.03.063Lad, H., Saumur, T. M., Herridge, M. S., Dos

Santos, C. C., Mathur, S., Batt, J., & Gilbert, P. M. (2020). Intensive Care Unit-Acquired Weakness: Not just Another Muscle Atrophying Condition. *International journal of molecular sciences*, 21(21), 7840. https://doi.org/10.3390/ijms21217840

Lancet, T. (2020). Editorial Facing up to long COVID. *The Lancet*, *396*(10266), 1861. https://doi.org/10.1016/S0140-6736(20)32662-3

Landi, F., Liperoti, R., Russo, A., Giovannini, S., Tosato, M., Capoluongo, E., Bernabei, R., & Onder, G. (2012). Sarcopenia as a risk factor for falls in elderly individuals: results from the ilSIRENTE study. *Clinical nutrition* (Edinburgh, Scotland), 31(5), 652–658. https://doi.org/10.1016/j.clnu.2012.02.007

Latronico, N., & Bolton, C. F. (2011). Critical illness polyneuropathy and myopathy: a major cause of muscle weakness and paralysis. *The Lancet. Neurology*, 10(10), 931– 941. https://doi.org/10.1016/S1474-4422(11)70178-8

Laughlin M. H. (1999). Cardiovascular response to exercise. *The American journal of physiology*, 277(6 Pt 2), S244–S259. https://doi.org/10.1152/advances.1999.277.6.S244

LaStayo, P. C., Marcus, R. L., Dibble, L. E., Smith, S. B., & Beck, S. L. (2011). Eccentric exercise versus usual-care with older cancer survivors: the impact on muscle and mobility--an exploratory pilot study. *BMC geriatrics*, 11, 5. https://doi.org/10.1186/1471-2318-11-5

LaStayo, P. C., Reich, T. E., Urquhart, M., Hoppeler, H., & Lindstedt, S. L. (1999). Chronic eccentric exercise: improvements in muscle strength can occur with little demand for oxygen. *The American journal of physiology*, 276(2), R611–R615. https://doi.org/10.1152/ajpregu.1999.276.2.R611

20

LaStayo, P., Marcus, R., Dibble, L., Frajacomo, F., & Lindstedt, S. (2014). Eccentric exercise in rehabilitation: safety, feasibility, and application. *Journal of applied physiology* (Bethesda, Md. : 1985), 116(11), 1426–1434. https://doi.org/10.1152/japplphysiol.00008.2013

LaStayo, P., Marcus, R., Dibble, L., Wong, B., & Pepper, G. (2017). Eccentric versus traditional resistance exercise for older adult fallers in the community: a randomized trial within a multi-component fall reduction program. *BMC geriatrics,* 17(1), 149. https://doi.org/10.1186/s12877-017-0539-8

LaStayo, P. C., Pierotti, D. J., Pifer, J., Hoppeler, H., & Lindstedt, S. L. (2000). Eccentric ergometry: increases in locomotor muscle size and strength at low training intensities. *American journal of physiology. Regulatory, integrative and comparative physiology*, 278(5), R1282–R1288. https://doi.org/10.1152/ajpregu.2000.278.5.R1282

LaStayo, P. C., Woolf, J. M., Lewek, M. D., Snyder-Mackler, L., Reich, T., & Lindstedt, S. L. (2003). Eccentric muscle contractions: their contribution to injury, prevention, rehabilitation, and sport. *The Journal of orthopaedic and sports physical therapy*, 33(10), 557–571. https://doi.org/10.2519/jospt.2003.33.10.557

Levine, S., Nguyen, T., Taylor, N., Friscia, M. E., Budak, M. T., Rothenberg, P., Zhu, J., Sachdeva, R., Sonnad, S., Kaiser, L. R., Rubinstein, N. A., Powers, S. K., & Shrager, J. B. (2008). Rapid disuse atrophy of diaphragm fibers in mechanically ventilated humans. *The New England journal of medicine*, 358(13), 1327–1335. https://doi.org/10.1056/NEJMoa070447

Ling, C. H., de Craen, A. J., Slagboom, P. E., Gunn, D. A., Stokkel, M. P., Westendorp, R. G., & Maier, A. B. (2011). Accuracy of direct segmental multi-frequency bioimpedance analysis in the assessment of total body and segmental body composition in middle-aged adult population. *Clinical nutrition* (Edinburgh, Scotland), 30(5), 610–615. https://doi.org/10.1016/j.clnu.2011.04.001

Liu, Y. C., Kuo, R. L., & Shih, S. R. (2020). COVID-19: The first documented coronavirus pandemic in history. *Biomedical journal*, 43(4), 328–333. https://doi.org/10.1016/j.bj.2020.04.007

Mak, I. W., Chu, C. M., Pan, P. C., Yiu, M. G., Ho, S. C., & Chan, V. L. (2010). Risk factors for chronic post-traumatic stress disorder (PTSD) in SARS survivors. *General hospital psychiatry*, 32(6), 590–598. https://doi.org/10.1016/j.genhosppsych.2010.07.007

Mandal, S., Barnett, J., Brill, S. E., Brown, J. S., Denneny, E. K., Hare, S. S.,

Heightman, M., Hillman, T. E., Jacob, J., Jarvis, H. C., Lipman, M., Naidu, S. B., Nair, A., Porter, J. C., Tomlinson, G. S., Hurst, J. R., & ARC Study Group (2020). 'Long COVID': a cross-sectional study of persisting symptoms, biomarker and imaging abnormalities following hospitalisation for COVID-19. *Thorax,* thoraxjnl-2020-215818. Advance online publication. https://doi.org/10.1136/thoraxjnl-2020-215818

Marcus, R. L., Smith, S., Morrell, G., Addison, O., Dibble, L. E., Wahoff-Stice, D., & Lastayo, P. C. (2008). Comparison of combined aerobic and high-force eccentric resistance exercise with aerobic exercise only for people with type 2 diabetes mellitus. *Physical therapy*, 88(11), 1345–1354. https://doi.org/10.2522/ptj.20080124

Massy-Westropp, N. M., Gill, T. K., Taylor, A. W., Bohannon, R. W., & Hill, C. L. (2011). Hand Grip Strength: age and gender stratified normative data in a population based study. *BMC research notes*, 4, 127. https://doi.org/10.1186/1756-0500-4-127

Mazza, M. G., De Lorenzo, R., Conte, C., Poletti, S., Vai, B., Bollettini, I., Melloni, E., Furlan, R., Ciceri, F., Rovere-Querini, P., COVID-19 BioB Outpatient Clinic Study group, & Benedetti, F. (2020). Anxiety and depression in COVID-19 survivors: Role of inflammatory and clinical predictors. *Brain, behavior, and immunity*, 89, 594–600. https://doi.org/10.1016/j.bbi.2020.07.037

Meyer, K., Steiner, R., Lastayo, P., Lippuner, K., Allemann, Y., Eberli, F., Schmid, J., Saner, H., & Hoppeler, H. (2003). Eccentric exercise in coronary patients: central hemodynamic and metabolic responses. *Medicine and science in sports and exercise*, 35(7), 1076–1082. https://doi.org/10.1249/01.MSS.0000074580.79648.9D

McCullough, P. A., Kelly, R. J., Ruocco, G., Lerma, E., Tumlin, J., Wheelan, K. R., Katz, N., Lepor, N. E., Vijay, K., Carter, H., Singh, B., McCullough, S. P., Bhambi, B. K., Palazzuoli, A., De Ferrari, G. M., Milligan, G. P., Safder, T., Tecson, K. M., Wang, D. D., McKinnon, J. E., … Risch, H. A. (2021). Pathophysiological Basis and Rationale for Early Outpatient Treatment of SARS-CoV-2 (COVID-19) Infection. *The American journal of medicine*, 134(1), 16–22. https://doi.org/10.1016/j.amjmed.2020.07.003

Mitchell, W. K., Taivassalo, T., Narici, M. V., & Franchi, M. V. (2017). Eccentric Exercise and the Critically Ill Patient. *Frontiers in physiology*, 8, 120. https://doi.org/10.3389/fphys.2017.00120

Musheyev, B., Borg, L., Janowicz, R., Matarlo, M., Boyle, H., Singh, G., Ende, V., Babatsikos, I., Hou, W., & Duong, T. Q. (2021). Functional status of mechanically ventilated COVID-19 survivors at ICU and hospital discharge. *Journal of intensive care*, 9(1), 31. https://doi.org/10.1186/s40560-021-00542-y

Nalbandian, A., Sehgal, K., Gupta, A., Madhavan, M. V., McGroder, C., Stevens, J.

S., Cook, J. R., Nordvig, A. S., Shalev, D., Sehrawat, T. S., Ahluwalia, N., Bikdeli, B., Dietz, D., Der-Nigoghossian, C., Liyanage-Don, N., Rosner, G. F., Bernstein, E. J., Mohan, S.,

Beckley, A. A., Seres, D. S., … Wan, E. Y. (2021). Post-acute COVID-19 syndrome. *Nature medicine*, 27(4), 601–615. https://doi.org/10.1038/s41591-021-

01283-z

Needham, D. M., Davidson, J., Cohen, H., Hopkins, R. O., Weinert, C., Wunsch, H., Zawistowski, C., Bemis-Dougherty, A., Berney, S. C., Bienvenu, O. J., Brady, S. L., Brodsky, M. B., Denehy, L., Elliott, D., Flatley, C., Harabin, A. L., Jones, C., Louis, D., Meltzer, W., Muldoon, S. R., … Harvey, M. A. (2012). Improving long-term outcomes after discharge from intensive care unit: report from a stakeholders' conference. *Critical care medicine*, 40(2), 502–509. https://doi.org/10.1097/CCM.0b013e318232da75

Nickel R, Troncoso F, Flores O, et al. Physiological response to eccentric and concentric cycling in patients with chronic obstructive pulmonary disease. *Applied Physiology, Nutrition, and Metabolism = Physiologie Appliquee, Nutrition et Metabolisme*. 2020 Nov;45(11):1232-1237. DOI: 10.1139/apnm-2020-0149.

Nunn, A. J., & Gregg, I. (1989). New regression equations for predicting peak expiratory flow in adults. *BMJ (Clinical research ed.),* 298(6680), 1068–1070. https://doi.org/10.1136/bmj.298.6680.1068

Pandharipande, P. P., Girard, T. D., Jackson, J. C., Morandi, A., Thompson, J. L., Pun, B. T., Brummel, N. E., Hughes, C. G., Vasilevskis, E. E., Shintani, A. K., Moons, K. G., Geevarghese, S. K., Canonico, A., Hopkins, R. O., Bernard, G. R., Dittus, R. S., Ely, E. W., & BRAIN-ICU Study Investigators (2013). Long-term cognitive impairment after critical illness. *The New England journal of medicine*, 369(14), 1306–1316. https://doi.org/10.1056/NEJMoa1301372

Paneroni, M., Simonelli, C., Saleri, M., Bertacchini, L., Venturelli, M., Troosters, T., Ambrosino, N., & Vitacca, M. (2021). Muscle Strength and Physical Performance in Patients Without Previous Disabilities Recovering From COVID-19 Pneumonia. *American journal of physical medicine & rehabilitation*, 100(2), 105–109. https://doi.org/10.1097/PHM.0000000000001641

Parshall, M. B., Schwartzstein, R. M., Adams, L., Banzett, R. B., Manning, H. L., Bourbeau, J., Calverley, P. M., Gift, A. G., Harver, A., Lareau, S. C., Mahler, D. A., Meek, P. M., O'Donnell, D. E., & American Thoracic Society Committee on Dyspnea (2012). An official American Thoracic Society statement: update on the mechanisms, assessment, and management of dyspnea. *American journal of respiratory and critical care medicine,* 185(4), 435–452. https://doi.org/10.1164/rccm.201111-2042ST

Patrocinio de Oliveira, C. E., Moreira, O. C., Carrión-Yagual, Z. M., Medina-Pérez,

C., & de Paz, J. A. (2018). Effects of Classic Progressive Resistance Training Versus Eccentric-Enhanced Resistance Training in People With Multiple Sclerosis. *Archives of physical medicine and rehabilitation*, 99(5), 819–825. https://doi.org/10.1016/j.apmr.2017.10.021

Peake, J. M., Suzuki, K., Hordern, M., Wilson, G., Nosaka, K., & Coombes, J. S. (2005). Plasma cytokine changes in relation to exercise intensity and muscle damage. *European journal of applied physiology*, 95(5-6), 514–521. https://doi.org/10.1007/s00421-005-0035-2

Peñailillo, L., Blazevich, A., & Nosaka, K. (2014). Energy expenditure and substrate oxidation during and after eccentric cycling. *European journal of applied physiology,* 114(4), 805–814. https://doi.org/10.1007/s00421-013-2816-3

Podsiadlo, D., & Richardson, S. (1991). The timed "Up & Go": a test of basic functional mobility for frail elderly persons. *Journal of the American Geriatrics Society*, 39(2), 142–148. https://doi.org/10.1111/j.1532-5415.1991.tb01616.x

Poulain, M., Durand, F., Palomba, B., Ceugniet, F., Desplan, J., Varray, A., & Préfaut, C. (2003). 6-minute walk testing is more sensitive than maximal incremental cycle testing for detecting oxygen desaturation in patients with COPD. *Chest*, *123*(5), 1401–1407. https://doi.org/10.1378/chest.123.5.1401

Ren C. L. (2003). What is the best way to measure lung function?. *Chest*, 123(3), 667–668. https://doi.org/10.1378/chest.123.3.667

Rocha Vieira, D. S., Baril, J., Richard, R., Perrault, H., Bourbeau, J., & Taivassalo, T. (2011). Eccentric cycle exercise in severe COPD: feasibility of application. *COPD,* 8(4), 270–274. https://doi.org/10.3109/15412555.2011.579926

Schuch, F. B., & Stubbs, B. (2019). The Role of Exercise in Preventing and Treating Depression. Current sports medicine reports, 18(8), 299–304.

https://doi.org/10.1249/JSR.0000000000000620

Shah, S., Vanclay, F., & Cooper, B. (1989). Improving the sensitivity of the Barthel Index for stroke rehabilitation. *Journal of clinical epidemiology*, 42(8), 703–709. https://doi.org/10.1016/0895-4356(89)90065-6

Shi, Y., Wang, Y., Shao, C., Huang, J., Gan, J., Huang, X., Bucci, E., Piacentini, M., Ippolito, G., & Melino, G. (2020). COVID-19 infection: the perspectives on immune responses. *Cell death and differentiation*, 27(5), 1451–1454. https://doi.org/10.1038/s41418-020-0530-3

Smith, L. L., Anwar, A., Fragen, M., Rananto, C., Johnson, R., & Holbert, D. (2000). Cytokines and cell adhesion molecules associated with high-intensity eccentric exercise. *European journal of applied physiology*, 82(1-2), 61–67. https://doi.org/10.1007/s004210050652

Smith, T., Gildeh, N., & Holmes, C. (2007). The Montreal Cognitive Assessment: validity and utility in a memory clinic setting. Canadian journal of psychiatry. *Revue canadienne de psychiatrie*, 52(5), 329–332. https://doi.org/10.1177/070674370705200508

Spies, C. D., Krampe, H., Paul, N., Denke, C., Kiselev, J., Piper, S. K., Kruppa, J., Grunow, J. J., Steinecke, K., Gülmez, T., Scholtz, K., Rosseau, S., Hartog, C., Busse, R., Caumanns, J.,

Marschall, U., Gersch, M., Apfelbacher, C., Weber-Carstens, S., & Weiss, B. (2021). Instruments to measure outcomes of post-intensive care syndrome in outpatient care settings - Results of an expert consensus and feasibility field test. *Journal of the Intensive Care Society*, 22(2), 159–174. https://doi.org/10.1177/1751143720923597

Ströhle, A., Schmidt, D. K., Schultz, F., Fricke, N., Staden, T., Hellweg, R., Priller, J., Rapp, M. A., & Rieckmann, N. (2015). Drug and Exercise Treatment of Alzheimer Disease and Mild Cognitive Impairment: A Systematic Review and Meta-Analysis of Effects on Cognition in Randomized Controlled Trials. *The American journal of geriatric psychiatry : official journal of the American Association for Geriatric Psychiatr*y, 23(12), 1234–1249. https://doi.org/10.1016/j.jagp.2015.07.007

Tansey, C. M., Louie, M., Loeb, M., Gold, W. L., Muller, M. P., de Jager, J., Cameron, J. I., Tomlinson, G., Mazzulli, T., Walmsley, S. L., Rachlis, A. R., Mederski, B. D., Silverman, M., Shainhouse, Z., Ephtimios, I. E., Avendano, M., Downey, J., Styra, R., Yamamura, D., Gerson, M., Herridge, M. S. (2007). One-year outcomes and health care utilization in survivors of severe acute respiratory syndrome. *Archives of internal medicine*, 167(12), 1312–1320. https://doi.org/10.1001/archinte.167.12.1312

Toft, A. D., Jensen, L. B., Bruunsgaard, H., Ibfelt, T., Halkjaer-Kristensen, J., Febbraio, M., & Pedersen, B. K. (2002). Cytokine response to eccentric exercise in young and elderly humans. *American journal of physiology*. Cell physiology, 283(1), C289–C295. https://doi.org/10.1152/ajpcell.00583.2001

Troyer, E. A., Kohn, J. N., & Hong, S. (2020). Are we facing a crashing wave of neuropsychiatric sequelae of COVID-19? Neuropsychiatric symptoms and potential immunologic mechanisms. *Brain, behavior, and immunity*, 87, 34–39. https://doi.org/10.1016/j.bbi.2020.04.027

Valenzuela JO, Gning I, Irarrázaval ME, Fasce G, Marín L, Mendoza TR, et al. Psychometric validation of the Spanish version of the Brief Fatigue Inventory [abstract]. The University of Texas MD Anderson Cancer Center, Division of Internal Medicine Research Retreat, Houston TX, May 24, 2012

Weiss, P., & Murdoch, D. R. (2020). Clinical course and mortality risk of severe COVID-19. *Lancet* (London, England), 395(10229), 1014–1015. https://doi.org/10.1016/S0140-6736(20)30633-4

Williamson, A., & Hoggart, B. (2005). Pain: a review of three commonly used pain rating scales. *Journal of clinical nursing*, 14(7), 798–804. https://doi.org/10.1111/j.1365-2702.2005.01121.x

Yamamoto, K., Takeshita, H., & Rakugi, H. (2020). ACE2, angiotensin 1-7 and skeletal muscle: review in the era of COVID-19. *Clinical science* (London, England : 1979), 134(22), 3047–3062. https://doi.org/10.1042/CS20200486

https://coronavirus.jhu.edu/map.html

https://coronavirus.mat.uc.cl/

https://data.gov.uk./

https://deis.minsal.cl/

https://www.gob.cl/coronavirus/cifrasoficiales/

https://www.who.int/es/emergencies/diseases/novel-coronavirus-2019

**Relevance of the Proposed Research**

**Indicate contribution to knowledge and other expected benefits.** *(Maximum 1000 words. Font: Arial – Size: 11)*

The global pandemic caused by SARS-CoV-2 has led to significant changes and consequences at all levels. The number of critically ill patients admitted to intensive care units in 2020 and 2021 had not been seen in years. Compared to 2019, ICU bed occupancy tripled in over 85% of hospitals in the country (DEIS, MINSAL). COVID-19 survivors—including not only severe cases but also moderate and even mild cases—continue to experience physical and psychological symptoms caused by the illness, many of which remain disabling (The Lancet, December 2020). There are still no official Chilean statistics on recovered ICU or hospitalized patients and their sequelae; however, the UK provides a reference: as of early June 2021, 376,000 people were recorded with post-COVID-19 symptoms and had not yet resumed their pre-illness lives (data.gov.uk). In a 2011 study by Herridge et al., about one-third of ICU survivors who were previously employed were unemployed 60 months after hospital discharge, coining the term “financial toxicity” due to the significant economic burden of recovering from a severe illness.

The pathophysiological complexity of the disease, including its multisystemic impact, results in the deterioration of various structures and functions that can lead to temporary or permanent disability in survivors (Mazza et al., 2020; Paneroni et al., 2021). The consequences for mostly young patients range widely—from fatigue, dyspnea, tiredness, dizziness, depression, anosmia, and cardiac disorders, among others—requiring a multidisciplinary rehabilitation program. However, the main issue is that most professionals capable of fulfilling these roles have been reassigned to acute care: hospitalized patients, home visits, epidemiological tasks, vaccination campaigns, etc. Therefore, there is an urgent need for a rehabilitation method that is both effective and efficient and can address multiple symptoms—otherwise, the social and health consequences for the country could be catastrophic.

There is evidence supporting the role of early rehabilitation. However, implementing exercise programs during ICU stays is often hindered by common features of critical illnesses such as circulatory instability due to sepsis, use of vasopressors, gas exchange impairment from ARDS and pneumonia, prone positioning, and limitations caused by IV lines and arterial catheters. Most critically, limited access for professionals and family members due to infection risk further hinders early rehabilitation therapy.

These limitations result in a major negative impact on the quality of life of both patients and their families, restricting basic and instrumental activities of daily living and limiting social participation. It is thus crucial to provide post-COVID-19 patients with an effective rehabilitation protocol that is tailored to the country’s realities and patient needs.

Current guidelines recommend functional rehabilitation therapy, but this often requires more than one therapist per patient. Many patients, having been bedridden for months, do not tolerate these protocols well. Tele-rehabilitation remains underdeveloped in the country—it often fails due to the inability to monitor vital signs remotely, the unfamiliarity of elderly or low-literacy patients with digital systems, and the limited internet access in rural areas.

This is why we propose a rehabilitation protocol using an eccentric arm and leg cycle ergometer, which allows patients to exercise both upper and lower limbs simultaneously while seated.

There is extensive literature on eccentric exercise and its benefits: it improves tissue elasticity, muscle strength, joint velocity, endurance, neuromuscular control, muscle fiber contraction capacity, proprioception, and concentric contraction response (LaStayo et al., 2003; Irby et al., 2020). It has also shown significant benefits in the physical recovery of patients with neurological conditions, including stroke (Engardt et al., 1995), and is well tolerated by those with severe heart failure (Casillas et al., 2016)—a condition also seen in COVID-19 survivors. Numerous studies have demonstrated that this form of exercise is safe and highly beneficial (LaStayo et al., 2013).

Based on this evidence, we propose a safe, resource-efficient, and well-tolerated rehabilitation method that leads to greater muscle gains with less cardiovascular demand—and also improves both physical and psychological health.

**Project Feasibility**

*(Maximum 1000 words. Font: Arial – Size: 11)*

The proposed work is a viable study. Patients will benefit regardless of whether they are assigned to the ECC group or the STD group, as both protocols are safe and will support their recovery. Due to the high hospital occupancy and staffing focused on acute care, rehabilitation—especially in the Los Lagos Region—is being neglected. Most patients have not undergone rehabilitation and have no access to it after recovering from the acute phase of the disease.

The physical space and most of the equipment will be provided by the **Nexus Center**, located in downtown Osorno, with easy transportation access. The center will participate in the research with no financial interest. The **InBody analyzer** and **dynamometer** will be provided by the Human Performance Department of the University of Los Lagos.

Initial laboratory tests for the study are already conducted as part of hospital discharge procedures. Since COVID-19 is covered under Chile’s GES (Explicit Health Guarantees), the healthcare system is responsible for covering related tests and follow-ups. All other evaluations will be performed at the Nexus Center by the lead researcher and staff.

Final admission to the study will be determined by a physician at the Nexus Center.

The cycle ergometer was purchased by the lead researcher.

**Work Plan – Timeline**

*(Maximum 1 page. Font: Arial – Size: 11)*

**Gantt Chart:** Attached in Excel format.

**Study Limitations**

- For safety reasons, it was not possible to perform standard pulmonary function or respiratory muscle tests such as spirometry, arterial blood gases, and diffusion capacity (DLCO). Health authorities issued guidance to limit aerosol-generating tests, meaning few centers offer them, and those that do have significantly increased their prices.
- Due to the novelty and rapid progression of the pandemic, there is still limited evidence regarding the rehabilitation of these patients. Specifically, there is no current evidence on **eccentric training in COVID-19 recovery**. Nor are there studies combining eccentric exercise with **mental health**, **cognitive impairment**, or **pulmonary fibrosis**—the latter being a known sequela of COVID-19.
- The scales used to evaluate dyspnea and fatigue are **subjective assessments**, influenced by each patient’s personal experience and tolerance, making comparisons difficult.
- The study sample will be **heterogeneous**: no differentiation will be made by age, sex, or comorbidities, which may influence the results of each protocol in individual patients.
- **Diet will not be monitored**, which could affect body composition changes and strength gains.
- Changes in COVID-19 control policies (e.g., transition phases of the “Step-by-Step” plan) may lead to renewed **quarantines**, which could hinder patient transportation to the rehabilitation center.
- Due to ongoing **viral circulation** and public health contact tracing protocols, if a patient or their caregiver is identified as a close contact or becomes infected, they must suspend protocol participation for **11 days**.

**III – ANNEXES**

**ANNEX I: Poster to recruit volunteers**

**ANNEX II: Guidelines for medical control for study admission**

**Identification:**

- Age
- Date of Contagion
- Date of Hospitalization
- Hospitalization Service and days
- Hospital Discharge
- Current employment situation
- Chronic Diseases/Surgeries
- Chronic Medications
- Habits (Alcohol, Tobacco, Drugs)
- Level of previous physical activity
- Family history

**VITAL SIGNS:**

- Temperature
- Blood Pressure
- Heart Rate
- Respiratory Rate
- Saturation

**PHYSICAL EXAMINATION:**

- Appearance, skin
- Cardiac auscultation
- Pulmonary auscultation
- Abdominal exploration

**NEUROLOGICAL EXPLORATION:**

- Cranial nerves
- Balance
- Orientation

**UPPER EXTREMITIES:**

- Strength
- Mobility
- Sensitivity
- Pulses

**LOWER EXTREMITIES:**

- Strength
- Mobility
- Sensitivity
- Pulses

**TESTS:**

- Imaging
- ECG

**LABORATORY TESTS:**

- Hemogram
- PCR (Polymerase Chain Reaction)
- ELP (Electrophoresis)
- Creatinine
- Lipid Profile
- Liver Profile
- Blood Glucose Level (Glycemia)
- Ferritin
- Coagulation Tests
- D-dimer
- Troponins
- Pro-BNP (B-type Natriuretic Peptide)
- CK (Creatine Kinase)

**Fit to participate:** YES NO

Altered D-dimer, troponins, Pro-BNP in the last 3 months cannot participate. Recommendations indicate absolute rest for 3 months if any of these variables are altered. The evaluated tests are those brought by the patient from hospitalization which are covered by the Emergency Law and the volunteer will not have to bear any cost of this evaluation. (https://www.chileatiende.gob.cl/fichas/2470-atencion-medica-en-unidades-de-urgencia-ley-de-urgencia)

**ANNEX III: Pain assessment scale**

**ANNEX IV: Medical Research Council (MRC) dyspnea assessment scale**

**ANNEX V: Arm and leg eccentric cycle ergometer**

- Arm and leg cycle ergometer, mechanical and automatic function (200 W)
- Company: Guangzhou Jiamei-Medical Technology Co LTD
- Model: ABJ-107-2

**ANNEX VI: STD group protocol**

- After warming up, 5 minutes of cycle ergometer will be performed at RPE of 6-10.

1. 3 sets x 8 repetitions: alternate in each session 2 upper limb exercises such as chest opening, rowing, bicep curl, tricep extension, at intermediate resistance, according to 9-11 RPE, according to patient tolerance and ability.
2. 3 sets x 8 repetitions: alternate in each session 2 exercises such as deadlift, squats, leg opening with elastic band, at intermediate resistance, according to 9-11 RPE, according to patient tolerance and ability.

**ANNEX VII: Borg Scale**

**ANNEX VIII: Medical Research Council Strength Scale**

**ANNEX IX: 6-Minute Walk Test**

- The test consists of measuring the distance a person can walk in 6 minutes as fast as possible. Dyspnea presence, heart rate, respiratory rate and oxygen saturation are evaluated at the beginning, immediately at the end of the test and after 5 minutes of recovery. If the test is a control, it should be performed at the same time as the previous one to minimize variability. The place should be a corridor 30 meters long, marked every 3 meters at the beginning, end and extremes. It should be performed in an environment with adequate temperature and pleasant atmosphere for the patient.
- **INSTRUCTIONS:**
  - The patient must rest for at least 10 minutes before the test.
  - Take and record patient's vital signs.
  - Explain and demonstrate the test to the patient.
  - Show and explain dyspnea and fatigue scale to the patient.
  - Set the timer for 6 minutes.
  - Verbally stimulate the patient every minute.
  - At the end of the test, immediately measure heart rate and respiratory rate, saturation, blood pressure, and magnitude on dyspnea and fatigue scales.
  - If during the test the patient presents; chest pain, intolerable dyspnea, cramp, sweating, cyanosis, pallor or any other discomfort, the test should be terminated and parameters recorded.
  - Re-measure parameters at 5 minutes after the test is completed.
  - Let the patient rest for 10 minutes and conclude the test.

**RELATIVE CONTRAINDICATIONS:**

- Heart rate > 120 per minute at rest.
- Systolic blood pressure > 180 mmHg.
- Diastolic blood pressure > 100 mmHg.
- Oxygen saturation at rest < 89%.

**6-Minute Walk Test Report**

- Identification Date
- Base
- Final
- Recovery
- Heart Rate
- Respiratory Rate
- Saturation
- Blood Pressure
- Dyspnea
- Fatigue
- Symptoms during or after the test
- Age: Weight: Height:
- Did you stop before 6 minutes? Reason.
- Meters walked: LLN: Desaturation: Decrease of at least 4% of baseline saturation for one minute.

**ANNEX X: 1 min Sit to Stand Test**

- After about 10 minutes of rest, the subject is asked to stand up from a chair as many times as possible in one minute. The number of times achieved and oxygen saturation are measured. It is important to choose a chair without wheels and without armrests, and a safe place so that the subject does not fall while performing the procedure. It should be stopped if the subject feels chest pain, intense cough, blurred vision or saturation less than 85%.

**ANNEX XI: BARTHEL INDEX**

**ANNEX XII: Post COVID-19 Functional Status Scale**

**ANNEX XIII: PHQ-9 Questionnaire**

- Major depressive syndrome (MDS): presence of 5 or more of the 9 depressive symptoms with a severity index of "more than half of the days" (≥2), and one of the symptoms being depressed mood or anhedonia.
- Other depressive syndrome (ODS): presence of two, three or four depressive symptoms for "more than half of the days" (≥2), and one of the symptoms being depressed mood or anhedonia.
- Positive depressive symptoms (SD+): presence of at least one or two depressive symptoms but does not meet previous criteria.

**ANNEX XIV: Maximum Expiratory Flow Technique with Wright Flowmeter**

1. Subject in standing or sitting position.
2. Must perform maximum inspiration.
3. Place mouthpiece in mouth.
4. Secure well with lips.
5. Exhale as hard and fast as possible.

- The maneuver must be repeated at least three times allowing adequate rest time between them.
- The two highest values must have a difference less than 20 L/min between them. If not achieved, subject must continue forced expiration maneuvers up to a maximum of 8 times.
- Record highest value and time of measurements.
- Always use Gregg and Nunn normal values chart or best known value if previously performed.

**ANNEX XV: Westropp Tables**

**ANNEX XVI: Balance assessment with Berg Scale**

1. **FROM SITTING TO STANDING POSITION**
   - INSTRUCTIONS: Please stand up. Try not to use your hands.
     - ( ) 4 able to stand up without using hands and stabilize independently
     - ( ) 3 able to stand up independently using hands
     - ( ) 2 able to stand up using hands and after several attempts
     - ( ) 1 needs minimal help to stand up or stabilize
     - ( ) 0 needs moderate to maximum assistance to stand up
2. **STANDING WITHOUT HELP**
   - INSTRUCTIONS: Please stand for two minutes without holding on.
     - ( ) 4 able to stand for 2 minutes safely
     - ( ) 3 able to stand for 2 minutes with supervision
     - ( ) 2 able to stand for 30 seconds without holding on
     - ( ) 1 needs several attempts to stand for 30 seconds without holding on
     - ( ) 0 unable to stand for 30 seconds without assistance
3. **SITTING WITHOUT BACK SUPPORT, BUT WITH FEET ON THE FLOOR OR ON A STOOL**
   - INSTRUCTIONS: Please sit with your arms at your sides for 2 minutes.
     - ( ) 4 able to sit safely for 2 minutes
     - ( ) 3 able to sit for 2 minutes under supervision
     - ( ) 2 able to sit for 30 seconds
     - ( ) 1 able to sit for 10 seconds
     - ( ) 0 unable to sit without help for 10 seconds
4. **FROM STANDING TO SITTING**
   - INSTRUCTIONS: Please sit down.
     - ( ) 4 sits safely with minimal use of hands
     - ( ) 3 controls descent using hands
     - ( ) 2 uses back of thighs against chair to control descent
     - ( ) 1 sits independently but does not control descent
     - ( ) 0 needs help to sit
5. **TRANSFERS**
   - INSTRUCTIONS: Prepare chairs for a pivot transfer. Ask the patient to first move to a seat with armrests and then to a seat without armrests. Two chairs (one with and one without armrests) or a bed and a chair can be used.
     - ( ) 4 able to transfer safely with minimal use of hands
     - ( ) 3 able to transfer safely with help of hands
     - ( ) 2 able to transfer with verbal instructions and/or supervision
     - ( ) 1 needs one person to assist
     - ( ) 0 needs two people to assist or supervise transfer to be safe
6. **STANDING WITHOUT HELP WITH EYES CLOSED**
   - INSTRUCTIONS: Please close your eyes and stand for 10 seconds.
     - ( ) 4 able to stand for 10 seconds safely
     - ( ) 3 able to stand for 10 seconds with supervision
     - ( ) 2 able to stand for 3 seconds
     - ( ) 1 unable to keep eyes closed for 3 seconds but able to stand firm
     - ( ) 0 needs help to avoid falling
7. **STANDING WITHOUT HOLDING ON WITH FEET TOGETHER**
   - INSTRUCTIONS: Please put your feet together and stand without holding on.
     - ( ) 4 able to stand with feet together safely and independently for 1 minute
     - ( ) 3 able to stand with feet together independently for 1 minute with supervision
     - ( ) 2 able to stand with feet together independently but unable to maintain position for 30 seconds
     - ( ) 1 needs help to achieve posture but able to stand for 15 seconds with feet together
     - ( ) 0 needs help to achieve posture and unable to maintain it for 15 seconds
8. **REACHING FORWARD WITH OUTSTRETCHED ARM WHILE STANDING**
   - INSTRUCTIONS: Raise arm to 90°. Stretch fingers and reach forward as far as possible. Examiner places a ruler at the end of fingers when arm is at 90°. Fingers should not touch ruler while reaching forward. Measure distance finger reaches while subject leans forward. When possible, ask patient to use both arms to avoid trunk rotation.
     - ( ) 4 able to lean forward comfortably >25 cm
     - ( ) 3 able to lean forward safely >12 cm

**ANNEX XVII: Brief Fatigue Inventory (BFI)**

An arithmetic average of all items is calculated, giving a total score of 1-10; 0 means "no fatigue"; 1-3: "mild"; 4-6: "moderate"; and 7-10: "severe".

**ANNEX XVIII: Montreal Cognitive Assessment (MoCA)**

**ANNEX XIX: Authorization Letter from the Director of Nexus Center**
