# Supplementary material for: Eccentric cycling is superior to standard rehabilitation for Post-ICU recovery in COVID-19 survivors
Source: PLoS One. 2026 Feb 6;21(2):e0340965. doi: 10.1371/journal.pone.0340965 (PMC12880636; doi:10.1371/journal.pone.0340965)
Supplement: S3 File — (DOCX) [file pone.0340965.s003.docx]

**PROYECTO DE INVESTIGACION**

**I - PRESENTACIÓN**

| Título del Proyecto **“Efectos del entrenamiento excéntrico sobre la funcionalidad y calidad de vida de pacientes post hospitalización de COVID-19 en unidades críticas: un estudio piloto ”** |
| --- |

| **Nombre**  **Estudiante** | Maria Fernanda Miranda Núñez | | |
| --- | --- | --- | --- |
| **Teléfono** | 9 34589032 | **Casilla Electrónica (e-mail)** | miranda.mariafernanda@gmail.com |

| **Área Disciplinaria** | | | |
| --- | --- | --- | --- |
| Rehabilitación |  | Ciencias del ejercicio | X |

| **Profesor Guía** |
| --- |
| Mauricio Venegas |

1

**III – PROPUESTA DE INVESTIGACIÓN**

| **Resumen del Proyecto**  Abstract del Proyecto (Máximo 500 palabras. Letra Arial – Tamaño 11, espaciado 1.5) |
| --- |
| En diciembre del año 2019, las autoridades de salud de China notificaron una enfermedad respiratoria infecto contagiosa asociada a un nuevo coronavirus (SARS-CoV-2), el cual rápidamente se disemino por todo el mundo provocando la pandemia que estamos viviendo. La infección respiratoria causada por este virus ha sido denominada COVID-19; de sus siglas en inglés; COronaVIrus Disease 2019. En la mayor parte de los casos genera una enfermedad leve e incluso asintomática, pero cerca de un 20% de los afectados requerirá de hospitalización en distintos grados de complejidad; los pacientes graves pueden permanecer semanas, incluso meses en unidades de cuidado intensivo (UCI). Las intervenciones agudas empleadas en el tratamiento de estos pacientes, como la ventilación mecánica, la sedación y el reposo prolongado en cama, producen importantes secuelas, a las que en conjunto se conocen como “Sindrome Post Cuidados Intensivos” o PICS por sus siglas en inlges. Las secuelas de un internamiento crítico pueden permanecer hasta 5 años posterior al alta y son multisistemicas; involucran el componente motor; atrofia muscular, neuropatías de extremidades superiores e inferiores, daño neuromuscular (debilidad adquirida en UCI), daño cognitivo y deterioro psicológico, provocando una importante discapacidad en el paciente que sobrevive esta hospitalización.  Siendo el promedio de estadia en UCI bajo ventilación mecánica de 21 dias en pacientes COVID 19 grave, es de eseperar un gran impacto en todas las esferas de la calidad de vida de estos sobrevivientes.  Al ser el COVID-19 una enfermedad nueva, las necesidades de rehabilitación de las personas graves que sobreviven sólo se pueden anticipar a partir de los datos existentes sobre la población general que ha recibido cuidados críticos y de datos de sobrevivientes del virus SARS y MERS que causan una patología muy semejante, aun así, existe poca literatura sobre la rehabilitación de estos pacientes.  Existe amplia evidencia del beneficio del entrenamiento excéntrico en patologías como la Enfermedad Pulmonar Cronica (EPOC), la insuficiencia cardiaca, el accidente vascular, la enfermedad de Parkinson y otras patologías como también beneficios en adultos mayores, este |

2

| tipo de entrenamiento se caracteriza por tener mayores ganancias de fuerza y masa muscular con el 50% del gasto metabólico, resultando en una mayor tolerancia al esfuerzo físico de parte del paciente.  A continuación, se propone un protocolo de rehabilitación en cicloergometro excéntrico de brazos y piernas para pacientes sobrevivientes de COVID-19 grave, el cual se espera induzca mayores aumentos de fuerza, mejore la capacidad física, impacte en las secuelas cognitivas y de salud mental, contribuyendo a la mejora en la calidad de vida de los sobrevivientes de COVID-19 grave  en comparación a un programa de rehabilitación estándar de kinesiología. |
| --- |

| **Objetivo General**. (Letra Arial – Tamaño 11, espaciado 1.5) |
| --- |
| Determinar el efecto de un protocolo de rehabilitación excéntrico sobre la recuperación física y calidad de vida en pacientes sobrevivientes de COVID 19 hospitalizados en unidades críticas, y compararlo con un protocolo de rehabilitación estándar de kinesiología. |

| **Objetivos Específicos**  (Máximo 1 página. Letra Arial – Tamaño 11, espaciado 1.5) |
| --- |
| ∙ Determinar el cambio del estado funcional, con la escala de estado funcional Post COVID-19 y comparar el resultado entre el entrenamiento excéntrico y un protocolo de rehabilitación estándar de kinesiología.  ∙ Determinar y comparar el cambio en la calidad de vida del entrenamiento excéntrico y un protocolo de rehabilitación estándar de kinesiología.  ∙ Determinar y comparar los cambios de composición corporal (masa y grasa) antropométricos de los participantes antes y después del entrenamento excéntrico y un protocolo de rehabilitación estándar de kinesiología.  ∙ Determinar y comparar el nivel de dependencia con el índice de Barthel, antes y después del entrenamiento excéntrico y un protocolo de rehabilitación estándar de kinesiología. ∙ Determinar cual protocolo logra mejorar los síntomas de disnea y fatiga. |

3

| ∙ Determinar y comparar los cambios en la capacidad cognitiva con el test de MoCA entre el entrenamiento excéntrico y un protocolo de rehabilitación estándar de kinesiología. ∙ Comparar las ganancias de fuerza de extremidades superiores e inferiores con el entrenamiento excéntrico y un protocolo de rehabilitación estándar de kinesiología.  ∙ Determinar el riesgo de caídas y equilibrio de los pacientes en el entrenamiento excéntrico y un protocolo de rehabilitación estándar de kinesiología.  ∙ Determinar y comparar los cambios en el flujo espiratorio máximo con flujometro de Wright del grupo con entrenamiento excéntrico y el grupo que realice un protocolo de rehabilitación estándar de kinesiología. |
| --- |

| **Fundamentos Teóricos**  (Introducción, marco teórico, y justificación del estudio, Máximo 3000 palabras. Letra Arial – Tamaño 11, espaciado 1.5) |
| --- |
| El 31 de diciembre de 2019 en la ciudad de Wuhan, China comenzó la alerta de la aparición de un nuevo virus que generaba neumonía y falla respiratoria catastrófica, el cual se disperso rapidamente por todo el mundo (Organización Mundial de la Salud, 2020). A la fecha (Julio 2021) se han contabilizado más de 60 millones de personas infectadas y más de 2.100.000 fallecidos en 189 países (Universidad de John Hopkins), en Chile, se han documentado 1.650.483 casos totales y 32.973 muertes (DEIS, Minsal).  El nuevo coronavirus; SARS-CoV-2, se transmite de persona en persona, su capacidad infectiva es muy alta, produce una gran cantidad de síntomas que varían desde un cuadro respiratorio alto, lesiones en piel, síntomas gastrointestinales, perdida del gusto y el olfato, a un cuadro de diestres respiratorio con inflamación sistémica grave, produciendo fenómenos tromboticos, vasculitis, miocarditis entre otras manifestaciones (McCullough et al, 2021). Alrededor de un 80% de los pacientes desarrolla un cuadro plurisintomatico leve mientras que el 20% restante desarrolla un cuadro clínico caracterizado por una neumonía intersticial bilateral que lleva al fallo respiratorio (Weiss & Murdoch, 2020; McCullough et al, 2021).  El virus en el organismo entra a las células utilizando la enzima convertidora de angiotensina 2 (ACE2), la cual se encuentra presente en muchas células del organismo; riñón, pulmones, corazón, mucosa nasal, hígado, vasos sanguíneos, sistema inmunológico, la piel, los músculos esqueléticos |

4

y el cerebro (Hoffman et al, 2020). Es por esto que las manifestaciones clínicas de la enfermedad son tan diversas. El virus tiene la capacidad de producir una respuesta inmunológica descarriada denominada “cascada citocínica”, la responsable del deterioro de los pacientes afectados con COVID-19 (Chen et al, 2020).

El uso del ventilador combinado con la sedación; el uso de relajantes musculares, las drogas vaso activas y períodos prolongados de inmovilización, agregado al uso de posición prono, tienen efectos negativos sobre todo el organismo, generando un sin número de complicaciones asociadas a la estadía en Unidades de Cuidados Intensivos que se las designó con el término de Síndrome Post

Cuidados Intensivos.Estas deficiencias persisten más allá de la hospitalización en la unidad de cuidados intensivos, y pueden durar de 5 a 15 años (Desai et al, 2011). Los componentes de este síndrome se pueden dividir en tres:

1. Complicaciones psicológicas: Los síntomas de ansiedad pueden llegar a afectar al 48% de los pacientes post UCI (Mazza et al, 2020), la depresión afectará cerca del 30% y el Trastorno por estrés postraumático (TEPT) entre 10 y 50% (Hodgson, 2017).

2. Complicaciones cognitivas: La disfunción cerebral en UCI es una de las formas más habituales y se manifiesta como delirium. En el período post-alta, se presenta a través de deterioro neurocognitivo; la atención, la memoria, la velocidad de procesamiento y las funciones ejecutivas son las más afectadas. Diferentes estudios han demostrado que el deterioro neurocognitivo afecta entre el 20 y el 80% de los sobrevivientes de la UCI (Pandharipande et al, 2013).

3. Complicaciones físicas: Pueden clasificarse en pulmonares y extrapulmonares. En las pulmonares, lo más frecuente de encontrar es un patrón restrictivo por la fibrosis pulmonar y debilidad de los músculos respiratorios (Levine et al, 2008; Fan et al, 2014), la cual se suma a las secuelas de fibrosis pulmonar del mismo COVID-19 que alteran la difusión de gases (Mc Donald, 2021) y en las extrapulmonares, se destaca el severo daño muscular denominado debilidad adquirida en UCI (UCI-AW), se define como la debilidad muscular aguda de las extremidades superiores e inferiores en un patrón simétrico, que es causada por una enfermedad crítica (Lad et al, 2020, Latronico et al, 2011). La incidencia de UCI ‐ AW es del 40% en pacientes adultos críticamente enfermos (Appleton, 2014). Los mecanismos fisiopatológicos de UCI ‐ AW son multifactoriales; el catabolismo y la inmovilidad provocan desgaste del músculo esquelético, mientras que las lesiones microvasculares (efecto secundario de las drogas vasoactivas) provocan isquemia en los nervios periféricos (Kress

5

2014). Al expresarse ACE2 en las células musculares, estas también están afectadas por el virus, siendo la musculatura lisa y la estriada un órgano blanco del SARS-CoV-2 (Yamamoto et al, 2020). El riesgo de UCI ‐ AW se asocia con sepsis, estado catabólico, falla multiorgánica, síndrome de respuesta inflamatoria sistémica, ventilación mecánica de larga duración, inmovilidad, hiperglucemia, glucocorticoides y agentes bloqueadores neuromusculares, la mayoría de estas variables las encontramos en el paciente COVID-19 (Appleton, 2015).

El PICS ahora se reconoce como una carga para la salud pública debido a la discapacidad funcional asociada. Sin embargo, su prevalencia exacta sigue siendo desconocida por ser una entidad nueva y subdiagnosticada. En circunstancias sin pandemia el PICS afecto del 30 al 50% de los pacientes sobrevivientes de unidades de cuidados intensivos (Delgado, 2017; Beaudart et al, 2018). Aproximadamente un tercio de los pacientes no regresa al trabajo, otro tercio de los pacientes no regresa a su mismo trabajo o al mismo salario previo a la hospitalización en UCI (Griffiths, 2013).

De los componentes del PICS; la UCI ‐ AW, están relacionada con una disminución de la calidad de vida, disminución de la capacidad física y aumento de la morbilidad-mortalidad (Beaudart et al, 2018, Fan 2014). Afecta del 26-65% de los pacientes que llevan más de 5 días con ventilación mecánica, tiene una relación directa entre el número de días bajo ventilación mecánica y la prevalencia de la enfermedad (Levine et al, 2008). En un estudio observacional de Tansey et al, 2007, sobre pacientes sobrevivientes de UCI de SARS, se reportó que todos los que fueron ventilados mecánicamente (117 pacientes) informaron debilidad y desgaste muscular en el momento del alta hospitalaria, y el 37% seguía informando una reducción importante en su salud física 1 año después de la hospitalización por cuidados agudos. En otro estudio de Hui et al, 2005, el rendimiento del test de marcha de 6 minutos (6MWDT) fue significativamente menor que el de la población normal al año después de la hospitalización. En un estudio transversal de Paneroni et al, 2021, se evaluó la fuerza del músculo cuádriceps y bíceps y el rendimiento físico en pacientes en recuperación de neumonía COVID-19 y encontraron que la contracción voluntaria máxima para los cuádriceps fue de un 54% y para los bíceps de un 69% del valor normal previsto. El rendimiento físico evaluado con la prueba “1min sit-to-stand test” (1minSTST), fue de un 63% del valor normal previsto. Al final de esta prueba, el 24% de los pacientes mostró desaturación inducida por el ejercicio. En otro estudio reciente se evaluó el estado funcional de 118 pacientes que estuvieron con ventilación mecánica con el índice de Barthel, de ellos el 85%

6

| era dependiente al alta hospitalaria, siendo que, al ingreso, el 94% era independiente (Musheyev et al, 2021). La alta prevalencia de deterioro de la fuerza y el rendimiento físico en estos pacientes sugiere la necesidad de programas de rehabilitación urgente. A todo esto, se agregan otros numerosos síntomas como la disnea a bajos esfuerzos, tos seca, fatiga, síntomas por disautonomia (palpitaciones, sincope, lipotimias, perdida de equilibrio, entre otros) cefalea, perdida del cabello, alteración del gusto y el olfato, y varios más (Mandal et al, 2020; Dani et al, 2021).  La rehabilitación motora es una estrategia clave en la recuperación de los pacientes después de una enfermedad crítica (Needham et al, 2012; Kress et al, 2014; Colbenson et al, 2019; Paneroni et al 2021). El PICS post COVID-19, necesitará de un manejo multidisciplinario incluyendo las intervenciones basadas en el ejercicio que tienen como objetivo mejorar la capacidad física, y  secundariamente pueden ayudar en el estado emocional y cognitivo del paciente, y podrían mejorar varios de los síntomas que persisten en los sobrevivientes.  Las diversas sociedades científicas de rehabilitación han puesto en evidencia la necesidad implementar programas para la fase post hospitalaria de la enfermedad, y aumentar la disponibilidad y la accesibilidad de los servicios de rehabilitación, pero la pandemia ha provocado que la mayoría de los recursos se enfoquen en tratar a los pacientes en estado crítico, dejando de lado la fase post-aguda.  Es urgente la necesidad de un trabajo de rehabilitación eficiente, fácil de aplicar para el personal de salud y de aprender para el paciente.  En un artículo de Gurovich, et al 2021 se planteó el entrenamiento con ejercicio excéntrico como una alternativa terapéutica para mejorar de manera eficiente el estado de salud física y mental de los pacientes post COVID-19, basado en evidencia de los beneficios de este entrenamiento en población clínica. Ademas, en una revisión de Mitchell et al, 2017, proponen el entrenamiento excéntrico seria la mejor opción para sobrevivientes de unidades críticas. El entrenamiento excéntrico se basa en la acción muscular “excéntrica”; existen tres tipos de acciones musculares; la concéntrica se produce cuando la tensión total generada produce un acortamiento muscular, la isométrica cuando la longitud del músculo permanece constante y la excéntrica cuando el músculo en lugar de contraerse aumenta su longitud. Ejemplos de trabajo excéntrico serian caminar cuesta abajo o bajar una escalera, trabajos que la mayoría de las personas siente “más ligeros”, pero el trabajo excéntrico es el estímulo |
| --- |

7

| más poderoso para aumentar la fuerza muscular y la movilidad a bajo costo metabólico (Lastayo et al, 1999; 2003).  Por lo tanto, el ejercicio excéntrico es un enfoque terapéutico novedoso que mejora la fuerza y la masa muscular con una demanda metabólica menor que el entrenamiento aeróbico convencional (Peñailillo et al, 2014). Ademas el ejercicio excéntrico se caracteriza por tener una mayor capacidad de generar fuerza y masa muscular a un menor costo metabólico (Lastayo et al, 1999), por lo que pareciera ser una alternativa ideal para ser utilizado en la restauración de la función del músculo esquelético en pacientes con PICS por COVID-19.  La aplicación del entrenamiento excéntrico se ha investigado en la rehabilitación de afecciones musculoesqueléticas (Irby et al 2020), en patologías crónicas como el EPOC (Nickel et al, 2020), en la insuficiencia cardíaca (Casillas et al, 2016), en la enfermedad de las arterias coronarias (Steiner et al, 2004), en diabetes tipo 2 (Marcus et al 2008), en sobrevivientes de cáncer (Lastayo et al, 2011), enfermedad de Parkinson (Bauer et al, 2018), esclerosis múltiple (Patrocinio de Oliveira et al, 2018) y en pacientes que sufrieron un accidente cerebrovascular (Engardt et al, 1995). Adicionalmemte se ha demostrado que los pacientes con EPOC grave toleran muy bien el cicloergometro excéntrico de intensidad progresiva, sin efectos secundarios (Rocha et al, 2011). En la insuficiencia cardiaca, el entrenamiento excéntrico a bajas tasas de esfuerzo percibido también es bien tolerado (Casillas et al, 2016). Por lo tanto, parece ser que la propuesta de un entrenamiento excéntrico en cicloergometro es una excelente alternativa para los pacientes sobrevivientes de COVID-19 quienes persisten con síntomas que les dificultan la actividad física como la disnea, la fatiga, la debilidad muscular generalizada (Mandal et al, 2020; Dani et al, 2021).  Por todo lo presentado anteriormente se propone un estudio piloto de 8 semanas de intervención en el cual se comparará la efectividad de un entrenamiento excéntrico versus un protocolo de rehabilitación estándar establecido por las guías de las distintas sociedades de rehabilitación, sobre la funcionalidad y calidad de vida de pacientes sobrevivientes de COVID-19 grave. |
| --- |

8

| **Metodología**  (Máximo 3000 palabras. Letra Arial – Tamaño 11, espaciado 1.5) |
| --- |
| Se realizará un estudio prospectivo comparativo aleatorizado con pacientes voluntarios que hayan estado hospitalizados por COVID-19 en alguna unidad de cuidados intensivos (previos 6 meses) y no hayan tenido rehabilitación posterior al alta hospitalaria.  Se incluirá a 20 pacientes voluntarios de la ciudad de Osorno y alrededores de la X Región de Los Lagos, previo a firma de consentimiento informado, convocados por afiches (Anexo I) en los mismos servicios hospitalarios, redes sociales y periódicos locales. El calculo del tamaño de la muestra fue calculado en base a los cambios de masa musucular observados en un estudio previo, considerando un 4.5% de aumento de masa muscular en pacientes EPOC después de 12 semanas de entrenamiento excéntrico (Peñailillo et al. 2021, In Press). Este calculo consideró un nivel de alfa de 0.05, 80% de poder estadistico y un P<0.05.  Criterio de inclusión: edad de los participantes de los 30-60 años. Todos los pacientes serán incluidos posterior a un chequeo médico general (Anexo II) realizado por la investigadora responsable de la investigacion.  Ciretrios de exclusión: pacientes que no completen las mediciones iniciales, durante el estudio y al final del mismo, quienes no estén aptos según chequeo médico (pacientes con miocarditis, troponinas o electrocardiogramas de los tres últimos meses alterados (Alderighi et al, 2020; Mahumud et al, 2020; Mckinney et al, 2020; Agustine et al, 2021), oxigeno dependientes o que tengan lesiones osteomusculares que le impidan la actividad, quienes tengan patologías que contraindiquen la actividad física) quienes no toleren la primera sesión para familiarizarse con el protocolo, pacientes desorientados, pacientes con discapacidad mental severa, pacientes postrados.  Todos los participantes deben firmar el consentimiento informado.  Ambos grupos realizaran una interrvencion por 8 semanas y se les realizaran mediciones antes y al finalizar ambas intervenciones. Durante las sesiones de entrenamiento se realizarán mediciones del monitoreo de signos vitales; presión arterial, saturación, frecuencia cardiaca, frecuencia respiratoria, dolor muscular de piernas y brazos con la escala evaluación análoga del dolor; EVA (Anexo III) y evaluacion de la disnea con la Escala de Disnea MRC (Anexo IV) antes y al finalizar cada sesión. Los participantes serán divididos en dos grupos aleatorizadamente, un grupo realizara el entrenamiento en el cicloergometro excéntrico (Anexo V) de brazos y piernas (Grupo ECC; n=10), el otro grupo realizara el protocolo de rehabilitacion estandar (Grupo STD; n=10), recomendado por el Consenso Interdisciplinario de rehabilitación para personas adultas post COVID-19 que consta |

9

de cicloergometro concéntrico y fortalecimiento de grupos musculares de extremidades superiores e inferiores con bandas elásticas y, o peso corporal (Anexo VI) . Ambos grupos realizaran una sesion previa al inicio del entrenamiento para familiarizarse con los equipos, ejercicios, escalas a medir y evaluar molestias posteriores a la sesión de actividad. Ambos grupos tendrán dos sesiones a la semana las primeras dos semanas (espaciadas por dos días) y luego tendrán 3 sesiones por

semana (espaciadas por un día) por un total de 8 semanas.

La intensidad del ejercicio se medirá con la escala de Borg de RPE (Rating of Perceived Exertion; Esfuerzo Percibido) que va del 6 al 20 donde 6 es “ningún esfuerzo en absoluto o reposo” y 20 sería el esfuerzo máximo (Anexo VII). Esta escala esta validada para su uso en población clínica, deportistas, niños y adultos mayores (Coquart et al, 2012).

Ambos grupos realizaran ejercicios de estiramiento y activación a modo de calentamiento previo de 5 minutos.

El grupo ECC, comenzará con dos ciclos de 10 minutos cada uno, separados por 2 minutos de descanso, a una escala de 9-11 RPE. De las semanas 3 a la 8 serán ciclos de 15 minutos separados por 2 minutos de descanso, a una escala de 13-15 RPE.

El grupo STD realizará un trabajo inicial de 5 minutos de cicloergometro a RPE de 6-10, seguido por 3 series x 8 repeticiones, de bandas elásticas (intermedia resistencia, según 9-11 RPE), con pausas de 2 minutos entre series; ejercicios para extremidades superiores y ejercicios para extremidades inferiores (ejemplo; para brazo, apertura de pectorales, remo, para piernas sentadillas, peso muerto).

Si alguno de los voluntarios presenta alguno de los siguientes sintomas no podrá realizar la actividad y su inicio se pospondrá según evaluación médica:

∙ Disnea intensa que no cede con reposo

∙ Dolor u opresión torácica

∙ Dificultad para respirar

∙ Tos intensa

∙ Mareos

∙ Visión borrosa

∙ Frecuencia cardiaca en reposo > 120 latidos por minuto

∙ Presión arterial en reposo < 80/60 mmHg o > 150/100 mmHg

∙ Saturación de oxígeno en sangre < 90% previo al ejercicio.

∙ Fiebre o síntomas de reinfección

10

**Las mediciones que se realizarían antes y despues de las 8 semanas de intervención para comparar los protocolos serán:**

∙ **Fuerza muscular (Medical Research Council) (Anexo VIII):** La escala MRC es una escala validada y fácil de utilizar, que permite evaluar la fuerza muscular en 3 grupos musculares de cada extremidad superior e inferior, en un rango de 0 (parálisis) a 5 (fuerza normal), para cada grupo muscular. El resultado final obtenido oscila entre 0 (parálisis total) y 60 (fuerza muscular normal en las 4 extremidades). Un valor por debajo de 48 se considera definitorio

para UCI-AW (Appleton, 2015). Las funciones evaluadas son: extensión de muñeca, flexión del codo, abducción del hombro, dorsiflexión de tobillo, extensión de rodilla, flexión de cadera.

∙ **Test de Marcha de 6 minutos (Anexo IX):** Ha demostrado ser una herramienta muy útil en la evaluación de la capacidad funcional de población clínica, nació como una alternativa al Test de Cooper; que evalúa la condición física en deportistas, en 1982 por Butland, y permite objetivar y realizar seguimiento de la rehabilitación en pacientes con limitación moderada a severa al ejercicio. El resultado se interpreta con la ecuación de Enright (Enright y Sherrill, 1998). Es muy práctico de aplicar en un ambiente clínico y de muy bajo costo y es considerado valido para evaluar el deterioro del intercambio gaseoso inducido por el ejercicio (Poulain et al, 2003; DuBois et al, 2011).

∙ **1 min Sit to Stand Test (Anexo X):** La prueba mide la cantidad de veces que el sujeto puede pararse de una silla y volver a sentarse sin ayuda de las extremidades superiores durante un minuto. Es una prueba fácil de realizar, barata y aporta información importante; se puede evaluar la fuerza de las extremidades inferiores (Bohannon, 1995), la desaturación de oxigeno durante la actividad física (Birand et al, 2018) y se puede medir el impacto de la rehabilitación (Vaifya et al, 2016), haciéndola una prueba alternativa para quienes no logren realizar el test de marcha de 6 minutos.

∙ **Actividades básicas de la vida diaria: Índice de Barthel (Anexo XI)**: Es un instrumento ampliamente utilizado para evaluar la capacidad de la persona para la realización de actividades básicas de la vida diaria, obteniéndose una estimación cuantitativa del grado de dependencia del sujeto. Se utiliza aproximadamente desde que fue propuesto en el año 1955 por Cid et al. Para evaluar a los pacientes se utilizará la modificación de Shah et al, con 10

11

| actividades que engloban tareas de aseo personal, comer, desplazarse en el domicilio, y 5 niveles de puntuación de 0; incapaz de hacerlo a 5; totalmente independiente. Se suman todos los ítems de 0-100, 0 totalmente dependiente a 100; totalmente independiente.  ∙ **Escala de estado funcional post COVID-19 (Anexo XII):** Este instrumento fue presentado en julio 2020 por Klock et al, traducida a más de 20 idiomas y es el único cuestionario validado hasta el momento para evaluar la funcionalidad de los pacientes sobrevivientes de COVID-19 al alta clinica. No está destinada a reemplazar otras herramientas de evaluación de actividades de la vida diaria, disnea, cansancio, etcétera, sino está hecha para complementar otras evaluaciones y medir objetivamente los avances en las terapias rehabilitadoras. Consta de una entrevista con preguntas son actividades de vida diaria, actividades instrumentales, actividades sociales y estilo de vida que el paciente debe calificar del 0 (sin limitaciones) al 4 (limitación funcional severa).  ∙ **Evaluación de síntomas depresivos con el Cuestionario PHQ-9 (Patient Health Questionnaire (Anexo XIII):** Es un instrumento desarrollado para la pesquisa de trastornos depresivos en atención primaria, tiene la ventaja de al ser una autoevaluación puede aplicarse a varios pacientes al mismo tiempo y se aplica en un corto tiempo (10 min aprox). Esta validado tanto para diagnostico como para seguimiento. Tiene una versión traducida para población chilena validada desde el 2012. Su caraceteristica principial es que no produce exacerbaciones ni síntomas disociativos en pacientes con patología depresiva  (Baader et al, 2012; Guía GES Depresión en personas de 15 años y más).  ∙ **Composicion corporal con InBody**: Se realizará una medición inicial de antropometría de cada paciente y una al final de los protocolos que incluye Peso (kg), Estatura (cm) y composición corporal (% grasa; % masa muscular) con InBody modelo 120 y software Lookin’Body120. Esta es una herramienta válida para las evaluaciones de la composición corporal total y segmentaria en la población general (Ling et al, 2011). Este dispositivo será facilitado para la investigación por el laboratorio de rendimiento humano de la Universidad de los Lagos. |
| --- |

12

∙ **Flujo espiratorio máximo con Flujometro (Anexo XIV):** Es una prueba de función pulmonar alternativa ante la ausencia de la espirometria (este es un examen generador de aerosoles y por la contingencia actual solo se está realizando en determinados casos y bajo estrictas condiciones sanitarias). La evaluación de la función pulmonar es obligatoria en las enfermedades respiratorias, donde este examen estará alterado ya sea por un aumento de la resistencia de la vía aérea, deterioro de la capacidad de difusión de los gases, o alteraciones en la mecánica respiratoria (Ren, 2003). El flujometro tiene una versión de uso personal (mini Wright), es un aparato portátil, de bajo costo, mide el máximo flujo espirado mantenido durante 10 milisegundos y que se expresa en litros por minuto, se basan en su capacidad de medir la resistencia calibrada que un resorte opone a la movilización de un émbolo al paso del aire y cuyo valor queda expresado en una escala visual. Los valores se comparan respecto a tablas estándar de Nunn y Gregg (Anexo XIV), además de comparar el resultado realizado en la primera evaluación respecto a evaluaciones posteriores. Es ampliamente utilizado en atención primaria en Chile desde la implementación de las Guías GES para asma y EPOC.

∙ **Evaluación de la fuerza con dinamómetro**: La fuerza de presión manual se ha considerado un indicador de fuerza global, tanto en población clínica como en deportistas (Cronin et al, 2017), y en pacientes con patologías cardiovasculares, respiratorias y adultos

mayores se ha relacionado con la mortalidad (Celis-Morales et al, 2018; Cooper et al, 2011).

Para medirla se utilizará un dinamómetro hidráulico de JAMAR ^®^, validado como

instrumento de medición (Hamilton et al, 1992). El paciente sentado con el codo en 90° debe

realizar una prensión máxima por 3 segundos, se realizarán 3 intentos, con un minuto de descanso, el mayor será el valor utilizado. Los resultados se compararán con las tablas de Westropp et al, 2011 (Anexo XV).

13

∙ **Disnea con MRCm (Medical Research Council modificada, Anexo IV):** La disnea es la sensación subjetiva de falta de aire; se origina de la integración de estímulos aferentes y eferentes de mecanoreceptores y quimioreceptores ubicados en distintas partes del organismo (Parshall et al, 2012). Durante el ejercicio aumentan las demandas ventilatorias en personas sanas y enfermas, apareciendo o aumentando así la disnea. Existen varias escalas para cuantificar la disnea, la escala de modificada Medical Research Council, es la más sencilla y fácil de aplicar.

∙ **Evaluación de riesgo de caídas con la prueba Timed up and go:** Es una prueba fácil de aplicar, se utiliza habitualmente para medir el riesgo de caídas

en adultos mayores (Lusardi et al 2017), y en varios estudios para evaluar pacientes sobrevivientes de UCI (Spies et al, 2021). Es una prueba confiable y válida para cuantificar la movilidad funcional, y para seguir el cambio clínico a lo largo del tiempo (Podsiadlo y Richardson, 1991). Se le pide al paciente que se siente en una silla, y al decirle “comience” se debe poner de pie y caminar 3 metros en línea recta, dar la vuelta y sentarse de nuevo. Se mide el tiempo en completar esta tarea, en segundos. El paciente debe realizar 3 intentos, estos se promedian para el cálculo total. Menos de 10 segundos se considera una persona independiente con bajo riesgo de caídas, entre 10-20 segundos; indica fragilidad, riesgo de caídas, más de 20 segundos; alto riesgo de caídas.

∙ **Evaluación del equilibrio con la Escala de Berg Anexo XVI:** También es una prueba utilizada para el riesgo de caídas por perdida del equilibrio (Lusardi et al 2017). Se ha utilizado en la evaluación de pacientes sobrevivientes de UCI (Smith et al, 2020; Spies et al, 2021). La prueba consta de 14 tareas a realizar y califica del 0 al 4 como se desempeña el paciente, el puntaje máximo es 56. De 0-20 puntos el paciente es totalmente dependiente, de 21 a 40 puede caminar con ayudas técnicas y entre 41-56 es una persona totalmente independiente, sin riesgo (Berg et al, 1989).

∙ **Evaluación de Fatiga con el inventario breve de fatiga; IBF Anexo XVII:** Es un síntoma predominante en los pacientes post COVID-19 y PICS (Kress et al 2014; Hodgson et al, 2017; Yamamoto et al, 2029). Existen múltiples escalas para evaluarla, el IBF es un cuestionario que evalúa el nivel de fatiga y su interferencia en las actividades de la vida

14

| diaria. Se evalúa del 0 al 10, siendo el 10 la mayor fatiga posible y mayor nivel de interferencia. Fue validad al español en el 2012 por Valenzuela et al y desde entonces es utilizada en enfermedades como Fibromialgia, Síndrome de Fatiga Crónica, Cáncer, entre otras enfermedades. Es corto, y fácil de comprender para los pacientes.  ∙ **Evaluación cognitiva de Montreal (MoCA) Anexo XVIII:** La evaluacion cognitiva de Montreal es una prueba cuya aplicacion lleva alrededor de 10 minutos y ayuda a pesquizar disfunciones cognitivas. Fue publicado el 2007 por un grupo que investigo durante varios pacientes de clinicas de memoria en Montreal (Smith et al, 2007). Evalua las areas; orientacion, memoria a corto plazo, funcionamiento visoespecial y ejecutiva, habilidades de lenguaje, abstraccion, denominacion de animales y atencion. Se suman todos los resultados, maximo es 30 puntos, se debe añadir un punto si la persona no tiene escolaridad. Bajo 26 puntos existe disfuncion cognitiva. Esta validado para los pacientes con PICS (Smith et al, 2020; Spies et al, 2021).  Los materiales y el lugar utilizado para el estudio será el Centro de Rehabilitación Nexus ubicado en la ciudad de Osorno, el director de la institución, Juan Eduardo Villagra acepto y firmo la carta de autorización para realizar el estudio en este centro (Anexo XIX).  **Plan de análisis estadístico**  Se realizará una comparación de las variables pre-post (medidas repetidas), utilizando un análisis de ANOVA de dos vías considerando 2 grupos y 2 tiempos de medición.  Para procesar los datos se utilizará el programa Excel de Microsoft Office y SPSS (Statistical Package for the Social Sciences). |
| --- |

| **Bibliografía**  (Mínimo 30 referencias, estilo Vancouver, Letra Arial – Tamaño 11) |
| --- |
| Appleton, R. T. D., Kinsella, J., & Quasim, T. (2015). The incidence of intensive care unit-acquired weakness syndromes: A systematic review. *Journal of the Intensive Care Society*, *16*(2), 126– 136. https://doi.org/10.1177/1751143714563016 |

15

Arbour, N., Day, R., Newcombe, J., & Talbot, P. J. (2000). Neuroinvasion by Human Respiratory Coronaviruses. *Journal of Virology*, *74*(19), 8913–8921. https://doi.org/10.1128/jvi.74.19.8913- 8921.2000

Baader M, Tomas, Molina F, José Luis, Venezian B, Silvia, Rojas C, Carmen, Farías S, Renata, Fierro-Freixenet, Carlos, Backenstrass, Mathias, & Mundt, Christoph. (2012). Validación y utilidad de la encuesta PHQ-9 (Patient Health Questionnaire) en el diagnóstico de depresión en pacientes usuarios de atención primaria en Chile. *Revista chilena de neuro psiquiatría*, 50(1), 22. https://dx.doi.org/10.4067/S0717-92272012000100002

Bauer, J., Vincent, I., Buckenmeyer, P., Sutherlin, M., Lind, E., Dames, K., Generali, A., Schrom, B., & Mills, M. (2017). Parkinson’s Disease: Eccentric Training To Reduce Symptoms 1299 June 1 8 00 AM - 8 20 AM. *Medicine & Science in Sports & Exercise*, *49*(5S), 352. https://doi.org/10.1249/01.mss.0000517843.91267.4a

Beaudart, C., Biver, E., Bruyère, O., Cooper, C., Al-Daghri, N., Reginster, J. Y., & Rizzoli, R. (2018). Assessment of Quality of Life in Musculo-Skeletal Health Europe PMC Funders Group. *Aging Clin Exp Res*, *30*(5), 413–418. https://doi.org/10.1007/s40520-017-0794-8.Assessment

Bohannon R. W. (1995). Sit-to-stand test for measuring performance of lower extremity muscles. *Perceptual and motor skills*, *80*(1), 163–166. https://doi.org/10.2466/pms.1995.80.1.163

Bourgonje, A. R., Abdulle, A. E., Timens, W., Hillebrands, J. L., Navis, G. J., Gordijn, S. J., Bolling, M. C., Dijkstra, G., Voors, A. A., Osterhaus, A. D., van der Voort, P. H., Mulder, D. J., & van Goor, H. (2020). Angiotensin-converting enzyme 2 (ACE2), SARS-CoV-2 and the pathophysiology of coronavirus disease 2019 (COVID-19). *The Journal of pathology,* 251(3), 228–248. https://doi.org/10.1002/path.5471

Butland, R. J., Pang, J., Gross, E. R., Woodcock, A. A., & Geddes, D. M. (1982). Two-, six-, and 12- minute walking tests in respiratory disease. *British medical journal (Clinical research ed.),* 284(6329), 1607–1608. https://doi.org/10.1136/bmj.284.6329.1607

Casillas, J. M., Besson, D., Hannequin, A., Gremeaux, V., Morisset, C., Tordi, N., et al. (2016). Effects of an eccentric training personalized by a low rate of perceived exertion on the maximal capacities in chronic heart failure: a randomized controlled trial. *Eur. J. Phys. Rehabil. Med.* 52, 159–168.

Celis-Morales, C. A., Welsh, P., Lyall, D. M., Steell, L., Petermann, F., Anderson, J., Iliodromiti, S., Sillars, A., Graham, N., Mackay, D. F., Pell, J. P., Gill, J., Sattar, N., & Gray, S. R. (2018). Associations of grip strength with cardiovascular, respiratory, and cancer outcomes and all cause mortality: prospective cohort study of half a million UK Biobank participants. BMJ (*Clinical research ed.*), 361, k1651. https://doi.org/10.1136/bmj.k1651

Chen, G., Wu, D., Guo, W., Cao, Y., Huang, D., Wang, H., Wang, T., Zhang, X., Chen, H., Yu, H., Zhang, X., Zhang, M., Wu, S., Song, J., Chen, T., Han, M., Li, S., Luo, X., Zhao, J., & Ning, Q. (2020). Clinical and immunological features of severe and moderate coronavirus disease 2019. *The Journal of clinical investigation,* 130(5), 2620–2629.

https://doi.org/10.1172/JCI137244

16

Cheng, S., & Wong, C. W. (2005). Psychological intervention with sufferers from severe acute respiratory syndrome (SARS): lessons learnt from empirical findings. *Clinical Psychology & Psychotherapy*, 12(1), 80–86. https://doi.org/10.1002/cpp.429

Cid-Ruzafa, Javier, & Damián-Moreno, Javier. (1997). Valoración de la discapacidad física: el indice de Barthel. *Revista Española de Salud Pública,* 71(2), 127-137. Recuperado en 20 de junio de 2021, de http://scielo.isciii.es/scielo.php?script=sci_arttext&pid=S1135- 57271997000200004&lng=es&tlng=es

Colbenson, G. A., Johnson, A., & Wilson, M. E. (2019). Post-intensive care syndrome: impact, prevention, and management. *Breathe* (Sheffield, England), 15(2), 98–101. https://doi.org/10.1183/20734735.0013-2019

Cooper, R., Kuh, D., Cooper, C., Gale, C. R., Lawlor, D. A., Matthews, F., Hardy, R., & FAL Con and HALCyon Study Teams (2011). Objective measures of physical capability and subsequent health: a systematic review. *Age and ageing*, 40(1), 14–23. https://doi.org/10.1093/ageing/afq117

Coquart, J. B., Tourny-Chollet, C., Lemaître, F., Lemaire, C., Grosbois, J. M., & Garcin, M. (2012). Relevance of the measure of perceived exertion for the rehabilitation of obese patients. *Annals of physical and rehabilitation medicine*, 55(9-10), 623–640. https://doi.org/10.1016/j.rehab.2012.07.003

Covid-19, A. P. (2020). Consenso Interdisciplinario de Rehabilitación para Personas. 1–154.

Cronin, J., Lawton, T., Harris, N., Kilding, A., & McMaster, D. T. (2017). A Brief Review of Handgrip Strength and Sport Performance. *Journal of strength and conditioning research,* 31(11), 3187–3217. https://doi.org/10.1519/JSC.0000000000002149

Dani, M., Dirksen, A., Taraborrelli, P., Torocastro, M., Panagopoulos, D., Sutton, R., & Lim, P. B. (2021). Autonomic dysfunction in 'long COVID': rationale, physiology and management strategies. *Clinical medicine* (London, England), 21(1), e63–e67.

https://doi.org/10.7861/clinmed.2020-0896

Desai, S. V., Law, T. J., & Needham, D. M. (2011). Long-term complications of critical care. *Critical care medicine*, 39(2), 371–379. https://doi.org/10.1097/CCM.0b013e3181fd66e5

Engardt, M., Knutsson, E., Jonsson, M., & Sternhag, M. (1995). Dynamic muscle strength training in stroke patients: effects on knee extension torque,

electromyographic activity, and motor function*. Archives of physical medicine and rehabilitation*, 76(5), 419–425. https://doi.org/10.1016/s0003-9993(95)80570-2

Enright, P. L., & Sherrill, D. L. (1998). Reference equations for the six-minute walk in healthy adults. *American journal of respiratory and critical care medicine*, 158(5 Pt 1), 1384–1387. https://doi.org/10.1164/ajrccm.158.5.9710086

Fan, E., Cheek, F., Chlan, L., Gosselink, R., Hart, N., Herridge, M. S., Hopkins, R. O., Hough, C. L., Kress, J. P., Latronico, N., Moss, M., Needham, D. M., Rich, M. M.,

17

Stevens, R. D., Wilson, K. C., Winkelman, C., Zochodne, D. W., Ali, N. A., ATS Committee on ICU-acquired Weakness in Adults, & American Thoracic Society (2014). An official American Thoracic Society Clinical Practice guideline: the diagnosis of intensive care unit-acquired weakness in adults*. American journal of respiratory and critical care medicine,* 190(12), 1437–1446.

Fan, E., Dowdy, D. W., Colantuoni, E., Mendez-Tellez, P. A., Sevransky, J. E., Shanholtz, C., Himmelfarb, C. R., Desai, S. V., Ciesla, N., Herridge, M. S., Pronovost, P. J., & Needham, D. M. (2014). Physical complications in acute lung injury survivors: a two-year longitudinal prospective study. *Critical care medicine*, 42(4), 849–859. https://doi.org/10.1097/CCM.0000000000000040

Flann, K. L., LaStayo, P. C., McClain, D. A., Hazel, M., & Lindstedt, S. L. (2011). Muscle damage and muscle remodeling: no pain, no gain?. *The Journal of experimental biology*, 214(Pt 4), 674–679. https://doi.org/10.1242/jeb.050112

Fridén, J., Seger, J., Sjöström, M., & Ekblom, B. (1983). Adaptive response in human skeletal muscle subjected to prolonged eccentric training. *International journal of sports medicine*, 4(3), 177–183. https://doi.org/10.1055/s-2008-1026031

Griffiths, J., Hatch, R. A., Bishop, J., Morgan, K., Jenkinson, C., Cuthbertson, B. H., & Brett, S. J. (2013). An exploration of social and economic outcome and associated health-related quality of life after critical illness in general intensive care unit survivors: a 12-month follow-up study. *Critical care* (London, England), 17(3), R100. https://doi.org/10.1186/cc12745

Guo, Q., Zheng, Y., Shi, J., Wang, J., Li, G., Li, C., Fromson, J. A., Xu, Y., Liu, X., Xu, H., Zhang, T., Lu, Y., Chen, X., Hu, H., Tang, Y., Yang, S., Zhou, H., Wang, X., Chen, H., Wang, Z., … Yang, Z. (2020). Immediate psychological distress in quarantined patients with COVID-19 and its association with peripheral inflammation: A mixed method study. *Brain, behavior, and immunity*, 88, 17–27. https://doi.org/10.1016/j.bbi.2020.05.038

Gurovich, Alvaro N. PT, PhD, FACSM1; Tiwari, Sangeeta PhD2; Kehl, Stephanie MS2; Umucu, Emre PhD3; Peñailillo, Luis PT, PhD4 A Novel “Eccentric” Therapeutic Approach for Individuals Recovering From COVID-19, *Cardiopulmonary Physical Therapy Journal:* April 2021 - Volume 32 - Issue - p S15-S21 doi: 10.1097/CPT.0000000000000163

Hamilton, G. F., McDonald, C., & Chenier, T. C. (1992). Measurement of grip strength: validity and reliability of the sphygmomanometer and jamar grip dynamometer. *The Journal of orthopaedic and sports physical therapy*, 16(5), 215–219. https://doi.org/10.2519/jospt.1992.16.5.215

18

Harrison, A. J., Burdon, C. A., Groeller, H., & Peoples, G. E. (2020). The Acute Physiological Responses of Eccentric Cycling During the Recovery Periods of a High Intensity Concentric Cycling Interval Session. *Frontiers in physiology*, 11, 336. https://doi.org/10.3389/fphys.2020.00336

Herridge, M. S., Tansey, C. M., Matté, A., Tomlinson, G., Diaz-Granados, N., Cooper, A., Guest, C. B., Mazer, C. D., Mehta, S., Stewart, T. E., Kudlow, P., Cook, D., Slutsky, A. S., Cheung, A. M., & Canadian Critical Care Trials Group (2011). Functional disability 5 years after acute respiratory distress syndrome. *The New England journal of medicine,* 364(14), 1293–1304. https://doi.org/10.1056/NEJMoa1011802

Hodgson, C. L., Udy, A. A., Bailey, M., Barrett, J., Bellomo, R., Bucknall, T., Gabbe, B. J., Higgins, A. M., Iwashyna, T. J., Hunt-Smith, J., Murray, L. J., Myles, P. S., Ponsford, J., Pilcher, D., Walker, C., Young, M., & Cooper, D. J. (2017). The impact of disability in survivors of critical illness. *Intensive care medicine*, 43(7), 992–1001. https://doi.org/10.1007/s00134-017-4830-0

Hoffmann, M., Kleine-Weber, H., Schroeder, S., Krüger, N., Herrler, T., Erichsen, S., Schiergens, T. S., Herrler, G., Wu, N. H., Nitsche, A., Müller, M. A., Drosten, C., & Pöhlmann, S. (2020). SARS-CoV-2 Cell Entry Depends on ACE2 and TMPRSS2 and Is Blocked by a Clinically Proven Protease Inhibitor. *Cell,* 181(2), 271–280.e8. https://doi.org/10.1016/j.cell.2020.02.052

Honigsbaum M. (2013). "An inexpressible dread": psychoses of influenza at fin-de siècle. *Lancet* (London, England), 381(9871), 988–989. https://doi.org/10.1016/S0140- 6736(13)60701-1

Hui, D. S., Wong, K. T., Ko, F. W., Tam, L. S., Chan, D. P., Woo, J., & Sung, J. J. (2005). The 1-year impact of severe acute respiratory syndrome on pulmonary function, exercise capacity, and quality of life in a cohort of survivors. *Chest*, 128(4), 2247–2261. https://doi.org/10.1378/chest.128.4.2247

Irby, A, Gutierrez, J, Chamberlin, C, Thomas, SJ, Rosen, AB. Clinical management of tendinopathy: A systematic review of systematic reviews evaluating the effectiveness of tendinopathy treatments. *Scand J Med Sci Sports*. 2020; 30: 1810– 1826. https://doi.org/10.1111/sms.13734

Kirk-Sanchez, N. J., & McGough, E. L. (2014). Physical exercise and cognitive performance in the elderly: current perspectives. *Clinical interventions in aging*, 9, 51– 62. https://doi.org/10.2147/CIA.S39506

Klok, F. A., Boon, G., Barco, S., Endres, M., Geelhoed, J., Knauss, S., Rezek, S. A.,

19

Spruit, M. A., Vehreschild, J., & Siegerink, B. (2020). The Post-COVID-19 Functional Status scale: a tool to measure functional status over time after COVID-19. *The European respiratory journal,* 56(1), 2001494. https://doi.org/10.1183/13993003.01494- 2020

Kress, J. P., & Hall, J. B. (2014). ICU-acquired weakness and recovery from critical illness. *The New England journal of medicine*, 370(17), 1626–1635. https://doi.org/10.1056/NEJMra1209390

Kvam, S., Kleppe, C. L., Nordhus, I. H., & Hovland, A. (2016). Exercise as a treatment for depression: A meta-analysis. Journal of affective disorders, 202, 67–86. https://doi.org/10.1016/j.jad.2016.03.063Lad, H., Saumur, T. M., Herridge, M. S., Dos Santos, C. C., Mathur, S., Batt, J., &

Gilbert, P. M. (2020). Intensive Care Unit-Acquired Weakness: Not just Another Muscle Atrophying Condition. *International journal of molecular sciences*, 21(21), 7840. https://doi.org/10.3390/ijms21217840

Lancet, T. (2020). Editorial Facing up to long COVID. *The Lancet*, *396*(10266), 1861. https://doi.org/10.1016/S0140-6736(20)32662-3

Landi, F., Liperoti, R., Russo, A., Giovannini, S., Tosato, M., Capoluongo, E., Bernabei, R., & Onder, G. (2012). Sarcopenia as a risk factor for falls in elderly individuals: results from the ilSIRENTE study. *Clinical nutrition* (Edinburgh, Scotland), 31(5), 652–658. https://doi.org/10.1016/j.clnu.2012.02.007

Latronico, N., & Bolton, C. F. (2011). Critical illness polyneuropathy and myopathy: a major cause of muscle weakness and paralysis. *The Lancet. Neurology*, 10(10), 931– 941. https://doi.org/10.1016/S1474-4422(11)70178-8

Laughlin M. H. (1999). Cardiovascular response to exercise. *The American journal of physiology*, 277(6 Pt 2), S244–S259. https://doi.org/10.1152/advances.1999.277.6.S244

LaStayo, P. C., Marcus, R. L., Dibble, L. E., Smith, S. B., & Beck, S. L. (2011). Eccentric exercise versus usual-care with older cancer survivors: the impact on muscle and mobility--an exploratory pilot study. *BMC geriatrics*, 11, 5. https://doi.org/10.1186/1471-2318-11-5

LaStayo, P. C., Reich, T. E., Urquhart, M., Hoppeler, H., & Lindstedt, S. L. (1999). Chronic eccentric exercise: improvements in muscle strength can occur with little demand for oxygen. *The American journal of physiology*, 276(2), R611–R615. https://doi.org/10.1152/ajpregu.1999.276.2.R611

20

LaStayo, P., Marcus, R., Dibble, L., Frajacomo, F., & Lindstedt, S. (2014). Eccentric exercise in rehabilitation: safety, feasibility, and application. *Journal of applied physiology* (Bethesda, Md. : 1985), 116(11), 1426–1434. https://doi.org/10.1152/japplphysiol.00008.2013

LaStayo, P., Marcus, R., Dibble, L., Wong, B., & Pepper, G. (2017). Eccentric versus traditional resistance exercise for older adult fallers in the community: a randomized trial within a multi-component fall reduction program. *BMC geriatrics,* 17(1), 149. https://doi.org/10.1186/s12877-017-0539-8

LaStayo, P. C., Pierotti, D. J., Pifer, J., Hoppeler, H., & Lindstedt, S. L. (2000). Eccentric ergometry: increases in locomotor muscle size and strength at low training intensities. *American journal of physiology. Regulatory, integrative and comparative physiology*, 278(5), R1282–R1288. https://doi.org/10.1152/ajpregu.2000.278.5.R1282

LaStayo, P. C., Woolf, J. M., Lewek, M. D., Snyder-Mackler, L., Reich, T., & Lindstedt, S. L. (2003). Eccentric muscle contractions: their contribution to injury, prevention, rehabilitation, and sport. *The Journal of orthopaedic and sports physical therapy*, 33(10), 557–571. https://doi.org/10.2519/jospt.2003.33.10.557

Levine, S., Nguyen, T., Taylor, N., Friscia, M. E., Budak, M. T., Rothenberg, P., Zhu, J., Sachdeva, R., Sonnad, S., Kaiser, L. R., Rubinstein, N. A., Powers, S. K., & Shrager, J. B. (2008). Rapid disuse atrophy of diaphragm fibers in mechanically ventilated humans. *The New England journal of medicine*, 358(13), 1327–1335. https://doi.org/10.1056/NEJMoa070447

Ling, C. H., de Craen, A. J., Slagboom, P. E., Gunn, D. A., Stokkel, M. P., Westendorp, R. G., & Maier, A. B. (2011). Accuracy of direct segmental multi-frequency bioimpedance analysis in the assessment of total body and segmental body composition in middle-aged adult population. *Clinical nutrition* (Edinburgh, Scotland), 30(5), 610–615. https://doi.org/10.1016/j.clnu.2011.04.001

Liu, Y. C., Kuo, R. L., & Shih, S. R. (2020). COVID-19: The first documented coronavirus pandemic in history. *Biomedical journal*, 43(4), 328–333. https://doi.org/10.1016/j.bj.2020.04.007

Mak, I. W., Chu, C. M., Pan, P. C., Yiu, M. G., Ho, S. C., & Chan, V. L. (2010). Risk factors for chronic post-traumatic stress disorder (PTSD) in SARS survivors. *General hospital psychiatry*, 32(6), 590–598. https://doi.org/10.1016/j.genhosppsych.2010.07.007

Mandal, S., Barnett, J., Brill, S. E., Brown, J. S., Denneny, E. K., Hare, S. S.,

21

Heightman, M., Hillman, T. E., Jacob, J., Jarvis, H. C., Lipman, M., Naidu, S. B., Nair, A., Porter, J. C., Tomlinson, G. S., Hurst, J. R., & ARC Study Group (2020). 'Long COVID': a cross-sectional study of persisting symptoms, biomarker and imaging abnormalities following hospitalisation for COVID-19. *Thorax,* thoraxjnl-2020-215818. Advance online publication. https://doi.org/10.1136/thoraxjnl-2020-215818

Marcus, R. L., Smith, S., Morrell, G., Addison, O., Dibble, L. E., Wahoff-Stice, D., & Lastayo, P. C. (2008). Comparison of combined aerobic and high-force eccentric resistance exercise with aerobic exercise only for people with type 2 diabetes mellitus. *Physical therapy*, 88(11), 1345–1354. https://doi.org/10.2522/ptj.20080124

Massy-Westropp, N. M., Gill, T. K., Taylor, A. W., Bohannon, R. W., & Hill, C. L. (2011). Hand Grip Strength: age and gender stratified normative data in a population based study. *BMC research notes*, 4, 127. https://doi.org/10.1186/1756-0500-4-127

Mazza, M. G., De Lorenzo, R., Conte, C., Poletti, S., Vai, B., Bollettini, I., Melloni, E., Furlan, R., Ciceri, F., Rovere-Querini, P., COVID-19 BioB Outpatient Clinic Study group, & Benedetti, F. (2020). Anxiety and depression in COVID-19 survivors: Role of inflammatory and clinical predictors. *Brain, behavior, and immunity*, 89, 594–600. https://doi.org/10.1016/j.bbi.2020.07.037

Meyer, K., Steiner, R., Lastayo, P., Lippuner, K., Allemann, Y., Eberli, F., Schmid, J., Saner, H., & Hoppeler, H. (2003). Eccentric exercise in coronary patients: central hemodynamic and metabolic responses. *Medicine and science in sports and exercise*, 35(7), 1076–1082. https://doi.org/10.1249/01.MSS.0000074580.79648.9D

McCullough, P. A., Kelly, R. J., Ruocco, G., Lerma, E., Tumlin, J., Wheelan, K. R., Katz, N., Lepor, N. E., Vijay, K., Carter, H., Singh, B., McCullough, S. P., Bhambi, B. K., Palazzuoli, A., De Ferrari, G. M., Milligan, G. P., Safder, T., Tecson, K. M., Wang, D. D., McKinnon, J. E., … Risch, H. A. (2021). Pathophysiological Basis and Rationale for Early Outpatient Treatment of SARS-CoV-2 (COVID-19) Infection. *The American journal of medicine*, 134(1), 16–22. https://doi.org/10.1016/j.amjmed.2020.07.003

Mitchell, W. K., Taivassalo, T., Narici, M. V., & Franchi, M. V. (2017). Eccentric Exercise and the Critically Ill Patient. *Frontiers in physiology*, 8, 120. https://doi.org/10.3389/fphys.2017.00120

Musheyev, B., Borg, L., Janowicz, R., Matarlo, M., Boyle, H., Singh, G., Ende, V., Babatsikos, I., Hou, W., & Duong, T. Q. (2021). Functional status of mechanically ventilated COVID-19 survivors at ICU and hospital discharge. *Journal of intensive care*, 9(1), 31. https://doi.org/10.1186/s40560-021-00542-y

Nalbandian, A., Sehgal, K., Gupta, A., Madhavan, M. V., McGroder, C., Stevens, J.

22



S., Cook, J. R., Nordvig, A. S., Shalev, D., Sehrawat, T. S., Ahluwalia, N., Bikdeli, B., Dietz, D., Der-Nigoghossian, C., Liyanage-Don, N., Rosner, G. F., Bernstein, E. J., Mohan, S., Beckley, A. A., Seres, D. S., … Wan, E. Y. (2021). Post-acute COVID-19 syndrome. *Nature medicine*, 27(4), 601–615. https://doi.org/10.1038/s41591-021-

01283-z

Needham, D. M., Davidson, J., Cohen, H., Hopkins, R. O., Weinert, C., Wunsch, H., Zawistowski, C., Bemis-Dougherty, A., Berney, S. C., Bienvenu, O. J., Brady, S. L., Brodsky, M. B., Denehy, L., Elliott, D., Flatley, C., Harabin, A. L., Jones, C., Louis, D., Meltzer, W., Muldoon, S. R., … Harvey, M. A. (2012). Improving long-term outcomes after discharge from intensive care unit: report from a stakeholders' conference. *Critical care medicine*, 40(2), 502–509. https://doi.org/10.1097/CCM.0b013e318232da75

Nickel R, Troncoso F, Flores O, et al. Physiological response to eccentric and concentric cycling in patients with chronic obstructive pulmonary disease. *Applied Physiology, Nutrition, and Metabolism = Physiologie Appliquee, Nutrition et Metabolisme*. 2020 Nov;45(11):1232-1237. DOI: 10.1139/apnm-2020-0149.

Nunn, A. J., & Gregg, I. (1989). New regression equations for predicting peak expiratory flow in adults. *BMJ (Clinical research ed.),* 298(6680), 1068–1070. https://doi.org/10.1136/bmj.298.6680.1068

Pandharipande, P. P., Girard, T. D., Jackson, J. C., Morandi, A., Thompson, J. L., Pun, B. T., Brummel, N. E., Hughes, C. G., Vasilevskis, E. E., Shintani, A. K., Moons, K. G., Geevarghese, S. K., Canonico, A., Hopkins, R. O., Bernard, G. R., Dittus, R. S., Ely, E. W., & BRAIN-ICU Study Investigators (2013). Long-term cognitive impairment after critical illness. *The New England journal of medicine*, 369(14), 1306–1316. https://doi.org/10.1056/NEJMoa1301372

Paneroni, M., Simonelli, C., Saleri, M., Bertacchini, L., Venturelli, M., Troosters, T., Ambrosino, N., & Vitacca, M. (2021). Muscle Strength and Physical Performance in Patients Without Previous Disabilities Recovering From COVID-19 Pneumonia. *American journal of physical medicine & rehabilitation*, 100(2), 105–109. https://doi.org/10.1097/PHM.0000000000001641

Parshall, M. B., Schwartzstein, R. M., Adams, L., Banzett, R. B., Manning, H. L., Bourbeau, J., Calverley, P. M., Gift, A. G., Harver, A., Lareau, S. C., Mahler, D. A., Meek, P. M., O'Donnell, D. E., & American Thoracic Society Committee on Dyspnea (2012). An official American Thoracic Society statement: update on the mechanisms, assessment, and management of dyspnea. *American journal of respiratory and critical care medicine,* 185(4), 435–452. https://doi.org/10.1164/rccm.201111-2042ST

Patrocinio de Oliveira, C. E., Moreira, O. C., Carrión-Yagual, Z. M., Medina-Pérez,

23

C., & de Paz, J. A. (2018). Effects of Classic Progressive Resistance Training Versus Eccentric-Enhanced Resistance Training in People With Multiple Sclerosis. *Archives of physical medicine and rehabilitation*, 99(5), 819–825. https://doi.org/10.1016/j.apmr.2017.10.021

Peake, J. M., Suzuki, K., Hordern, M., Wilson, G., Nosaka, K., & Coombes, J. S. (2005). Plasma cytokine changes in relation to exercise intensity and muscle damage. *European journal of applied physiology*, 95(5-6), 514–521. https://doi.org/10.1007/s00421-005-0035-2

Peñailillo, L., Blazevich, A., & Nosaka, K. (2014). Energy expenditure and substrate oxidation during and after eccentric cycling. *European journal of applied physiology,* 114(4), 805–814. https://doi.org/10.1007/s00421-013-2816-3

Podsiadlo, D., & Richardson, S. (1991). The timed "Up & Go": a test of basic functional mobility for frail elderly persons. *Journal of the American Geriatrics Society*, 39(2), 142–148. https://doi.org/10.1111/j.1532-5415.1991.tb01616.x

Poulain, M., Durand, F., Palomba, B., Ceugniet, F., Desplan, J., Varray, A., & Préfaut, C. (2003). 6-minute walk testing is more sensitive than maximal incremental cycle testing for detecting oxygen desaturation in patients with COPD. *Chest*, *123*(5), 1401–1407. https://doi.org/10.1378/chest.123.5.1401

Ren C. L. (2003). What is the best way to measure lung function?. *Chest*, 123(3), 667–668. https://doi.org/10.1378/chest.123.3.667

Rocha Vieira, D. S., Baril, J., Richard, R., Perrault, H., Bourbeau, J., & Taivassalo, T. (2011). Eccentric cycle exercise in severe COPD: feasibility of application. *COPD,* 8(4), 270–274. https://doi.org/10.3109/15412555.2011.579926

Schuch, F. B., & Stubbs, B. (2019). The Role of Exercise in Preventing and Treating Depression. Current sports medicine reports, 18(8), 299–304.

https://doi.org/10.1249/JSR.0000000000000620

Shah, S., Vanclay, F., & Cooper, B. (1989). Improving the sensitivity of the Barthel Index for stroke rehabilitation. *Journal of clinical epidemiology*, 42(8), 703–709. https://doi.org/10.1016/0895-4356(89)90065-6

Shi, Y., Wang, Y., Shao, C., Huang, J., Gan, J., Huang, X., Bucci, E., Piacentini, M., Ippolito, G., & Melino, G. (2020). COVID-19 infection: the perspectives on immune responses. *Cell death and differentiation*, 27(5), 1451–1454. https://doi.org/10.1038/s41418-020-0530-3

24



Smith, L. L., Anwar, A., Fragen, M., Rananto, C., Johnson, R., & Holbert, D. (2000). Cytokines and cell adhesion molecules associated with high-intensity eccentric exercise. *European journal of applied physiology*, 82(1-2), 61–67. https://doi.org/10.1007/s004210050652

Smith, T., Gildeh, N., & Holmes, C. (2007). The Montreal Cognitive Assessment: validity and utility in a memory clinic setting. Canadian journal of psychiatry. *Revue canadienne de psychiatrie*, 52(5), 329–332. https://doi.org/10.1177/070674370705200508

Spies, C. D., Krampe, H., Paul, N., Denke, C., Kiselev, J., Piper, S. K., Kruppa, J., Grunow, J. J., Steinecke, K., Gülmez, T., Scholtz, K., Rosseau, S., Hartog, C., Busse, R., Caumanns, J., Marschall, U., Gersch, M., Apfelbacher, C., Weber-Carstens, S., & Weiss, B. (2021). Instruments to measure outcomes of post-intensive care syndrome in outpatient care settings - Results of an expert consensus and feasibility field test. *Journal of the Intensive Care Society*, 22(2), 159–174. https://doi.org/10.1177/1751143720923597

Ströhle, A., Schmidt, D. K., Schultz, F., Fricke, N., Staden, T., Hellweg, R., Priller, J., Rapp, M. A., & Rieckmann, N. (2015). Drug and Exercise Treatment of Alzheimer Disease and Mild Cognitive Impairment: A Systematic Review and Meta-Analysis of Effects on Cognition in Randomized Controlled Trials. *The American journal of geriatric psychiatry : official journal of the American Association for Geriatric Psychiatr*y, 23(12), 1234–1249. https://doi.org/10.1016/j.jagp.2015.07.007

Tansey, C. M., Louie, M., Loeb, M., Gold, W. L., Muller, M. P., de Jager, J., Cameron, J. I., Tomlinson, G., Mazzulli, T., Walmsley, S. L., Rachlis, A. R., Mederski, B. D., Silverman, M., Shainhouse, Z., Ephtimios, I. E., Avendano, M., Downey, J., Styra, R., Yamamura, D., Gerson, M., Herridge, M. S. (2007). One-year outcomes and health care utilization in survivors of severe acute respiratory syndrome. *Archives of internal medicine*, 167(12), 1312–1320. https://doi.org/10.1001/archinte.167.12.1312

Toft, A. D., Jensen, L. B., Bruunsgaard, H., Ibfelt, T., Halkjaer-Kristensen, J., Febbraio, M., & Pedersen, B. K. (2002). Cytokine response to eccentric exercise in young and elderly humans. *American journal of physiology*. Cell physiology, 283(1), C289–C295. https://doi.org/10.1152/ajpcell.00583.2001

Troyer, E. A., Kohn, J. N., & Hong, S. (2020). Are we facing a crashing wave of neuropsychiatric sequelae of COVID-19? Neuropsychiatric symptoms and potential immunologic mechanisms. *Brain, behavior, and immunity*, 87, 34–39. https://doi.org/10.1016/j.bbi.2020.04.027

25



| Valenzuela JO, Gning I, Irarrázaval ME, Fasce G, Marín L, Mendoza TR, et al. Psychometric validation of the Spanish version of the Brief Fatigue Inventory [abstract]. The University of Texas MD Anderson Cancer Center, Division of Internal Medicine Research Retreat, Houston TX, May 24, 2012  Weiss, P., & Murdoch, D. R. (2020). Clinical course and mortality risk of severe COVID-19. *Lancet* (London, England), 395(10229), 1014–1015. https://doi.org/10.1016/S0140-6736(20)30633-4  Williamson, A., & Hoggart, B. (2005). Pain: a review of three commonly used pain rating scales. *Journal of clinical nursing*, 14(7), 798–804. https://doi.org/10.1111/j.1365-2702.2005.01121.x  Yamamoto, K., Takeshita, H., & Rakugi, H. (2020). ACE2, angiotensin 1-7 and skeletal muscle: review in the era of COVID-19. *Clinical science* (London, England : 1979), 134(22), 3047–3062. https://doi.org/10.1042/CS20200486  https://coronavirus.jhu.edu/map.html  https://coronavirus.mat.uc.cl/  https://data.gov.uk./  https://deis.minsal.cl/  https://www.gob.cl/coronavirus/cifrasoficiales/  https://www.who.int/es/emergencies/diseases/novel-coronavirus-2019 |
| --- |

| **Relevancia de la Investigación propuesta**.  Indicar aporte al conocimiento y otros beneficios esperados. (Máximo 1000 palabras. Letra Arial – Tamaño 11) |
| --- |
| La pandemia provocada por el SARS CoV-2 a nivel mundial ha producido una gran cantidad de cambios, secuelas en todos los niveles, hace años no se veía una cantidad de ingresos de pacientes graves en las unidades de paciente critico como en el año 2020 y 2021, en comparación al año 2019 la ocupación de camas UCI se ha triplicado en más del 85% de los hospitales del país (DEIS, Minsal). Los sobrevivientes de COVID-19, no solo los pacientes graves, sino pacientes de mediana |

26



gravedad, incluso pacientes con sintomatología leves persisten con síntomas físicos y psicológicos provocados por la misma enfermedad, los cuales en muchos casos son aun invalidantes (The Lancet, diciembre 2020). Aun no hay estadísticas oficiales chilenas de los pacientes recuperados de unidades UCI y hospitalización o de sus secuelas; pero como ejemplo tenemos las estadísticas del Reino Unido, quienes a principio de junio 2021 tenían contabilizadas 376,000 personas con síntomas post COVID-19, que aún no podían retornar a su vida antes de padecer la enfermedad (data.gov.uk). En un estudio de Herridge et al, 2011, aproximadamente un tercio de las personas sobrevivientes de la UCI previamente empleadas se encontraban cesantes a los 60 meses posteriores al alta hospitalaria, acuñándose el concepto de “toxicidad financiera”, dado el gran impacto económico que significa para una persona el haberse recuperado de una enfermedad grave.

La complejidad fisiopatológica de la enfermedad, incluyendo la afectación multisitémica sobre el organismo, implica el deterioro de diversas estructuras y funciones que pueden llevar a una situación de discapacidad transitoria o permanente en los sobrevivientes (Mazza et al, 2020; Paneroni et al, 2021). Las consecuencias del COVID-19 en estos pacientes, la gran mayoría pacientes jóvenes aun, son numerosas y heterogéneas; desde fatiga, disnea, cansancio, vértigo, depresión, anosmia, trastornos cardiacos, entre otros síntomas (Nalbandian et al, 2021) exigen la necesidad de un programa de rehabilitación multidisciplinario que abarque la gran diversidad de síntomas, pero la problemática está en que el personal dispuesto a estas funciones se encuentra en su gran mayoría reubicado para trabajar con pacientes agudos; pacientes hospitalizados en todos los servicios, visitas domiciliaras, labores epidemiológicas, proceso de vacunación, entre otras funciones, por lo que es urgente utilizar un método de rehabilitación efectivo y eficiente que pueda beneficiar varios sintomas, de lo contrario las secuelas socio sanitarias para el país serán catastróficas.

Existe evidencia que respalda el papel de la rehabilitación temprana, sin embargo, el cumplimiento de los programas de ejercicio durante la hospitalización en UCI puede verse reducido por características comunes de enfermedades críticas como la inestabilidad circulatoria asociada a la sepsis y administración de vasopresores, el intercambio de gases comprometido con el síndrome de dificultad respiratoria aguda y neumonía, la posición prono, la posición de las vías venosas y

catéteres arteriales, lo más importante, la limitación del ingreso a las salas de hospitalización de los profesionales y familiares por riesgo a infectarse se suman a la limitación para comenzar terapias de rehabilitación tempranas.

27



| Lo relatado anteriormente implica un impacto negativo enorme sobre la calidad de vida de la persona y su familia, limitando la realización de las actividades básicas e instrumentales de la vida diaria y restringiendo su participación en la sociedad. Se hace evidente, por lo tanto, la necesidad de entregar un protocolo de rehabilitación a las personas post COVID-19 eficaz y que se adecue a las realidades del país y a las necesidades de los pacientes.  Las guías actuales recomiendan o prescriben una terapia funcional para la rehabilitación, pero esta implica de más de un terapeuta asistiendo a cada paciente, los pacientes no toleran bien el protocolo, dado que muchos llevan meses de encamamiento, la alternativa de la tele rehabilitación aun es muy precaria en el país; no siempre resulta; no se pueden medir los signos vitales, los adultos mayores y pacientes de baja escolaridad no están familiarizados con este sistema, y hay muchos pacientes de áreas rurales, donde la conexión a internet es limitada.  Es por todo lo descrito que se propone un protocolo de rehabilitación en cicloergometro excéntrico de brazos y piernas, el cual el paciente lo realiza sentado trabajando extremidades superiores e inferiores a la vez.  Son numerosas las publicaciones sobre el ejercicio excéntrico y sus beneficios; en cuanto a la rehabilitación de lesiones, aumenta la elasticidad de los tejidos, aumenta la fuerza muscular y la velocidad articular, aumenta la fuerza-resistencia, mejora el control neuromuscular, mejora la capacidad de contracción de las fibras musculares, mejora la capacidad propioceptiva, mejora la respuesta de la contracción concéntrica (LaStayo et al, 2003; Irby et al, 2020). En pacientes con  enfermedades neurológicas, incluidas el accidente vascular, también ha mostrado multiples beneficios para la recuperación física de aquellos pacientes secuelados (Engardt et al 1995). Es bien tolerada en pacientes con grados severos de insuficiencia cardiaca (Casillas et al, 2016) patología que también se ve en pacientes sobrevivientes de COVID-19, y muchas patologías más. Está demostrado que es un ejercicio seguro y con múltiples beneficios (LaStayo et al, 2013).  Dado lo argumentado se propone de un método seguro de rehabilitación, con menos uso de recursos, bien tolerado, con mayor ganancia muscular a menos exigencia del sistema cardiopulmonar, y que además mejorara al paciente física y psicológicamente. |
| --- |

28

| **Viabilidad del Proyecto**  (Máximo 1000 palabras. Letra Arial – Tamaño 11) |
| --- |
| El trabajo propuesto es un estudio viable. Los pacientes tendrán beneficios ya sea les toque en el grupo ECC o en el grupo STD, son ambos protocolos seguros y ayudaran a su recuperación. Puesto el gran nivel de ocupación hospitalaria, el recurso de personal trabajando con pacientes agudos, la rehabilitación, sobre todo en la X región se está dejando de lado por lo que la mayoría de los pacientes no ha realizado rehabilitación y no tiene donde realizarla una vez superado el periodo agudo de la enfermedad.  El lugar físico y la mayoría de los implementos los facilitara el Centro Nexus, ubicado en el centro de la ciudad de Osorno, con fácil acceso a movilización, quienes participaran sin interés lucrativo en la investigación. El InBody y el dinamómetro será facilitado por el departamento de rendimiento humano de la Universidad de Los Lagos.  Los exámenes de laboratorio iniciales para el estudio vienen realizados desde su alta y controles hospitalarios, ya que al ser el COVID-19 incluido como una patología GES es obligación del sistema de salud costear exámenes y controles, las otras evaluaciones del estudio serán realizadas en el Centro Nexus por la investigadora y el personal del Centro.  El pase final al estudio será hecho por un médico del Centro Nexus.  El cicloergometro fue costeado por la investigadora. |

| **Plan de trabajo – Cronograma**  (Máximo 1 página. Letra Arial – Tamaño 11) |
| --- |
| **Carta Gantt:** Se adjunta en formato Excel. |

29

| **Limitaciones del estudio** |
| --- |
| ∙ Por razones de seguridad, fue imposible realizar pruebas estándar de función pulmonar o musculatura respiratoria, como la espirometria, gases arteriales y la evaluación de la capacidad de difusión (DLCO). La autoridad sanitaria emitió un comunicado de limitar todos los exámenes productores de aerosoles por lo que en muy pocos lugares realizan estos exámenes, y los lugares que lo realizan, elevaron mucho su costo.  ∙ Dado lo reciente y rápido de la pandemia, aun no hay gran cantidad de evidencia sobre la rehabilitación de estos pacientes, específicamente en trabajo excéntrico y COVID 19 aún no hay evidencia al respecto. Tampoco se encontraron investigaciones sobre ejercicio excéntrico y salud mental, daño cognitivo, ni trabajo excéntrico y fibrosis pulmonar, que sería lo más parecido con las secuelas pulmonares del COVID-19.  ∙ Las escalas utilizadas para la disnea y la fatiga son evaluaciones subjetivas, influenciadas por la vivencia personal y tolerancia de cada paciente, lo que hace difícil compararlas. ∙ El grupo será heterogéneo, no se distinguirá en edades, sexo o enfermedades crónicas que tengan los pacientes de base, lo que podrá afectar el resultado de cada protocolo en los pacientes.  ∙ No se supervisará la alimentación de los pacientes, lo que puede influir en los cambios antropométricos y ganancias de fuerza de los participantes.  ∙ Los cambios de “Fase” del plan “Paso a Paso” pueden producir que volvamos a cuarentena lo que puede comprometer el traslado de los pacientes al centro.  ∙ Dada la circulación viral y el protocolo de la autoridad sanitaria sobre los contactos estrechos, si los pacientes o quienes los trasladen son contactos estrechos o se contagian, tendrán que estar 11 días sin realizar el protocolo. |

30
